# Digital Interventions for Recreational Cannabis Use Among Young Adults: Systematic Review, Meta-Analysis, and Behavior Change Technique Analysis of Randomized Controlled Studies

**DOI:** 10.2196/55031

**Published:** 2024-04-17

**Authors:** José Côté, Gabrielle Chicoine, Billy Vinette, Patricia Auger, Geneviève Rouleau, Guillaume Fontaine, Didier Jutras-Aswad

**Affiliations:** 1 Faculty of Nursing Université de Montréal Montreal, QC Canada; 2 Research Centre of the Centre Hospitalier de l’Université de Montréal Montreal, QC Canada; 3 Research Chair in Innovative Nursing Practices Montreal, QC Canada; 4 Knowledge Translation Program Li Ka Shing Knowledge Institute St. Michael’s Hospital Toronto, ON Canada; 5 Department of Nursing Université du Québec en Outaouais Saint-Jérôme, QC Canada; 6 Women's College Hospital Institute for Health System Solutions and Virtual Care Women's College Hospital Toronto, ON Canada; 7 Ingram School of Nursing Faculty of Medicine and Health Sciences McGill University Montreal, QC Canada; 8 Centre for Clinical Epidemiology Lady Davis Institute for Medical Research Sir Mortimer B. Davis Jewish General Hospital Montreal, QC Canada; 9 Kirby Institute University of New South Wales Sydney Australia; 10 Department of Psychiatry and Addictology Faculty of Medicine Université de Montréal Montreal, QC Canada

**Keywords:** cannabis, young adults, digital intervention, systematic review, meta-analysis, behavior change technique analysis, mobile phone

## Abstract

**Background:**

The high prevalence of cannabis use among young adults poses substantial global health concerns due to the associated acute and long-term health and psychosocial risks. Digital modalities, including websites, digital platforms, and mobile apps, have emerged as promising tools to enhance the accessibility and availability of evidence-based interventions for young adults for cannabis use. However, existing reviews do not consider young adults specifically, combine cannabis-related outcomes with those of many other substances in their meta-analytical results, and do not solely target interventions for cannabis use.

**Objective:**

We aimed to evaluate the effectiveness and active ingredients of digital interventions designed specifically for cannabis use among young adults living in the community.

**Methods:**

We conducted a systematic search of 7 databases for empirical studies published between database inception and February 13, 2023, assessing the following outcomes: cannabis use (frequency, quantity, or both) and cannabis-related negative consequences. The reference lists of included studies were consulted, and forward citation searching was also conducted. We included randomized studies assessing web- or mobile-based interventions that included a comparator or control group. Studies were excluded if they targeted other substance use (eg, alcohol), did not report cannabis use separately as an outcome, did not include young adults (aged 16-35 y), had unpublished data, were delivered via teleconference through mobile phones and computers or in a hospital-based setting, or involved people with mental health disorders or substance use disorders or dependence. Data were independently extracted by 2 reviewers using a pilot-tested extraction form. Authors were contacted to clarify study details and obtain additional data. The characteristics of the included studies, study participants, digital interventions, and their comparators were summarized. Meta-analysis results were combined using a random-effects model and pooled as standardized mean differences.

**Results:**

Of 6606 unique records, 19 (0.29%) were included (n=6710 participants). Half (9/19, 47%) of these articles reported an intervention effect on cannabis use frequency. The digital interventions included in the review were mostly web-based. A total of 184 behavior change techniques were identified across the interventions (range 5-19), and *feedback on behavior* was the most frequently used (17/19, 89%). Digital interventions for young adults reduced cannabis use frequency at the 3-month follow-up compared to control conditions (including passive and active controls) by −6.79 days of use in the previous month (95% CI −9.59 to −4.00; *P*<.001).

**Conclusions:**

Our results indicate the potential of digital interventions to reduce cannabis use in young adults but raise important questions about what optimal exposure dose could be more effective, both in terms of intervention duration and frequency. Further high-quality research is still needed to investigate the effects of digital interventions on cannabis use among young adults.

**Trial Registration:**

PROSPERO CRD42020196959; https://www.crd.york.ac.uk/prospero/display_record.php?RecordID=196959

## Introduction

### Cannabis Use Among Young Adults Is Recognized as a Public Health Concern

Young adulthood (typically the ages of 18-30 y) is a critical developmental stage characterized by a peak prevalence of substance use [1,2]. Worldwide, cannabis is a substance frequently used for nonmedical purposes due in part to its high availability in some regions and enhanced product variety and potency [3,4]. The prevalence of cannabis use (CU) among young adults is high [5,6], and its rates have risen in recent decades [7]. In North America and Oceania, the estimated past-year prevalence of CU is ≥25% among young adults [8,9].

While the vast majority of cannabis users do not experience severe problems from their use [4], the high prevalence of CU among young adults poses substantial global health concerns due to the associated acute and long-term health and psychosocial risks [10,11]. These include impairment of cognitive function, memory, and psychomotor skills during acute intoxication; increased engagement in behaviors with a potential for injury and fatality (eg, driving under the influence); socioeconomic problems; and diminished social functioning [4,12-14]. Importantly, an extensive body of literature reveals that subgroups engaging in higher-risk use, such as intensive or repeated use, are more prone to severe and chronic consequences, including physical ailments (eg, respiratory illness and reproductive dysfunction), mental health disorders (eg, psychosis, depression, and suicidal ideation or attempts), and the potential development of CU disorder [4,15-17].

### Interventions to Reduce Public Health Impact of Young Adult CU

Given the increased prevalence of lifetime and daily CU among young adults and the potential negative impact of higher-risk CU, various prevention and intervention programs have been implemented to help users reduce or cease their CU. These programs primarily target young adults regardless of their CU status [2,18]. In this context, many health care organizations and international expert panels have developed evidence-based lower-risk CU guidelines to promote safer CU and intervention options to help reduce risks of adverse health outcomes from nonmedical CU [4,16,17,19]. Lower-risk guidance-oriented interventions for CU are based on concepts of health promotion [20-22] and health behavior change [23-26] and on other similar harm reduction interventions implemented in other areas of population health (eg, lower-risk drinking guidelines, supervised consumption sites and services, and sexual health) [27,28]. These interventions primarily aim to raise awareness of negative mental, physical, and social cannabis-related consequences to modify individual-level behavior-related risk factors.

Meta-analyses have shown that face-to-face prevention and treatment interventions are generally effective in reducing CU in young adults [18,29-32]. However, as the proportion of professional help seeking for CU concerns among young adults remains low (approximately 15%) [33,34], alternative strategies that consider the limited capacities and access-related barriers of traditional face-to-face prevention and treatment facilities are needed. Digital interventions, including websites, digital platforms, and mobile apps, have emerged as promising tools to enhance the accessibility and availability of evidence-based programs for young adult cannabis users. These interventions address barriers such as long-distance travel, concerns about confidentiality, stigma associated with seeking treatment, and the cost of traditional treatments [35-37]. By overcoming these barriers, digital interventions have the potential to have a stronger public health impact [18,38].

### State of Knowledge of Digital Interventions for CU and Young Adults

The literature regarding digital interventions for substance use has grown rapidly in the past decade, as evidenced by several systematic reviews and meta-analyses of randomized controlled trial (RCT) studies on the efficacy or effectiveness of these interventions in preventing or reducing harmful substance use [2,39-41]. However, these reviews do not focus on young adults specifically. In addition, they combine CU-related outcomes with those of many other substances in their meta-analytical results. Finally, they do not target CU interventions exclusively.

In total, 4 systematic reviews and meta-analyses of digital interventions for CU among young people have reported mixed results [42-45]. In their systematic review (10 studies of 5 prevention and 5 treatment interventions up to 2012), Tait et al [44] concluded that digital interventions effectively reduced CU among adolescents and adults at the posttreatment time point. Olmos et al [43] reached a similar conclusion in their meta-analysis of 9 RCT studies (2 prevention and 7 treatment interventions). In their review, Hoch et al [42] reported evidence of small effects at the 3-month follow-up based on 4 RCTs of brief motivational interventions and cognitive behavioral therapy (CBT) delivered on the web. In another systematic review and meta-analysis, Beneria et al [45] found that web-based CU interventions did not significantly reduce consumption. However, these authors indicated that the programs tested varied significantly across the studies considered and that statistical heterogeneity was attributable to the inclusion of studies of programs targeting more than one substance (eg, alcohol and cannabis) and both adolescents and young adults. Beneria et al [45] recommend that future work “establish the effectiveness of the newer generation of interventions as well as the key ingredients” of effective digital interventions addressing CU by young people. This is of particular importance because behavior change interventions tend to be complex as they consist of multiple interactive components [46].

Behavior change interventions refer to “coordinated sets of activities designed to change specified behavior patterns” [47]. Their interacting active ingredients can be conceptualized as behavior change techniques (BCTs) [48]. BCTs are specific and irreducible. Each BCT has its own individual label and definition, which can be used when designing and reporting complex interventions and as a nomenclature system when coding interventions for their content [47]. The Behavior Change Technique Taxonomy version 1 (BCTTv1) [48,49] was developed to provide a shared, standardized terminology for characterizing complex behavior change interventions and their active ingredients. Several systematic reviews with meta-regressions that used the BCTTv1 have found interventions with certain BCTs to be more effective than those without [50-53]. A better understanding of the BCTs used in digital interventions for young adult cannabis users would help not only to establish the key ingredients of such interventions but also develop and evaluate effective interventions.

In the absence of any systematic review of the effectiveness and active ingredients of digital interventions designed specifically for CU among community-living young adults, we set out to achieve the following:

1. conduct a comprehensive review of digital interventions for preventing, reducing, or ceasing CU among community-living young adults,
2. describe the active ingredients (ie, BCTs) in these interventions from the perspective of behavior change science, and
3. analyze the effectiveness of these interventions on CU outcomes.

## Methods

### Protocol Registration

We followed the Cochrane Handbook for Systematic Reviews of Interventions [54] in designing this systematic review and meta-analysis and the PRISMA (Preferred Reporting Items for Systematic Reviews and Meta-Analyses) 2020 guidelines in reporting our findings (see Multimedia Appendix 1 [55] for the complete PRISMA checklist). This review was registered in PROSPERO (CRD42020196959).

### Search Strategy

#### Overview

The search strategy was designed by a health information specialist together with the research team and peer reviewed by another senior information specialist before execution using Peer Review of Electronic Search Strategies for systematic reviews [56]. The search strategy revolved around three concepts:

1. CU (eg, “cannabis,” “marijuana,” and “hashish”)
2. Digital interventions (eg, “telehealth,” “website,” “mobile applications,” and “computer”)
3. Young adults (eg, “emerging adults” and “students”)

The strategy was initially implemented on March 18, 2020, and again on October 13, 2021, and February 13, 2023. The full, detailed search strategies for each database are presented in Multimedia Appendix 2.

#### Information Sources

We searched 7 electronic databases of published literature: CINAHL Complete, Cochrane Database of Systematic Reviews, Cochrane Central Register of Controlled Trials, Embase, MEDLINE, PubMed, and PsycINFO. No publication date filters or language restrictions were applied. A combination of free-text keywords and Medical Subject Headings was tailored to the conventions of each database for optimal electronic searching. The research team also manually screened the reference lists of the included articles and the bibliographies of existing systematic reviews [18,31,42-45] to identify additional relevant studies (snowballing). Finally, a forward citation tracking procedure (ie, searching for articles that cited the included studies) was carried out in Google Scholar.

#### Inclusion Criteria

The population, intervention, comparison, outcome, and study design process is presented in Multimedia Appendix 3. The inclusion criteria were as follows: (1) original research articles published in peer-reviewed journals; (2) use of an experimental study design (eg, RCT, cluster RCT, or pilot RCT); (3) studies evaluating the effectiveness (or efficacy) of digital interventions designed specifically to prevent, reduce, or cease CU as well as promote CU self-management or address cannabis-related harm and having CU as an outcome measure; (4) studies targeting young adults, including active and nonactive cannabis users; (5) cannabis users and nonusers not under substance use treatment used as controls in comparator, waitlist, or delayed-treatment groups offered another type of intervention (eg, pharmacotherapy or psychosocial) different from the one being investigated or participants assessed only for CU; and (6) quantitative CU outcomes (frequency and quantity) or cannabis abstinence. Given the availability of numerous CU screening and assessment tools with adequate psychometric properties and the absence of a gold standard in this regard [57], any instrument capturing aspects of CU was considered. CU outcome measures could be subjective (eg, self-reported number of CU days or joints in the previous 3 months) or objective (eg, drug screening test). CU had to be measured before the intervention (baseline) and at least once after.

Digital CU interventions were defined as web- or mobile-based interventions that included one or more activities (eg, self-directed or interactive psychoeducation or therapy, personalized feedback, peer-to-peer contact, and patient-to-expert communication) aimed at changing CU [58]. Mobile-based interventions were defined as interventions delivered via mobile phone through SMS text message, multimedia messaging service (ie, SMS text messages that include　multimedia content, such as pictures, videos, or emojis), or mobile apps, whereas web-based interventions (eg, websites and digital platforms) were defined as interventions designed to be accessed on the web (ie, the internet), mainly via computers. Interventions could include self-directed and web-based interventions with human support. We defined young adults as aged 16 to 35 years and included students and nonstudents. While young adulthood is typically defined as covering the ages of 18 to 30 years [59], we broadened the range given that the age of majority and legal age to purchase cannabis differs across countries and jurisdictions. This was also in line with the age range targeted by several digital CU interventions (college or university students or emerging adults aged 15-24 years) [31,45]. Given the language expertise of the research team members and the available resources, only English- and French-language articles were retained.

#### Exclusion Criteria

Knowledge synthesis articles, study protocols, and discussion papers or editorials were excluded, as were articles with cross-sectional, cohort, case study or report, pretest-posttest, quasi-experimental, or qualitative designs. Mixed methods designs were included only if the quantitative component was an RCT. We excluded studies if (1) use of substances other than cannabis (eg, alcohol, opioids, or stimulants) was the focus of the digital intervention (though studies that included polysubstance users were retained if CU was assessed and reported separately); (2) CU was not reported separately as an outcome or only attitudes or beliefs regarding, knowledge of, intention to reduce, or readiness or motivation to change CU was measured; and (3) the data reported were unpublished (eg, conferences and dissertations). Studies of traditional face-to-face therapy delivered via teleconference on mobile phones and computers or in a hospital-based setting and informational campaigns (eg, web-based poster presentations or pamphlets) were excluded as well. Studies with samples with a maximum age of <15 years and a minimum age of >35 years were also excluded. Finally, we excluded studies that focused exclusively on people with a mental health disorder or substance use disorder or dependence or on adolescents owing to the particular health care needs of these populations, which may differ from those of young adults [1].

### Data Collection

#### Selection of Studies

Duplicates were removed from the literature search results in EndNote (version X9.3.3; Clarivate Analytics) using the Bramer method for deduplication of database search results for systematic reviews [60]. The remaining records were uploaded to Covidence (Veritas Health Innovation), a web-based systematic review management system. A reviewer guide was developed that included screening questions and a detailed description of each inclusion and exclusion criterion based on PICO (population, intervention, comparator, and outcome), and a calibration exercise was performed before each stage of the selection process to maximize consistency between reviewers. Titles and abstracts of studies flagged for possible inclusion were screened first by 2 independent reviewers (GC, BV, PA, and GR; 2 per article) against the eligibility criteria (stage 1). Articles deemed eligible for full-text review were then retrieved and screened for inclusion (stage 2). Full texts were assessed in detail against the eligibility criteria again by 2 reviewers independently. Disagreements between reviewers were resolved through consensus or by consulting a third reviewer.

#### Data Extraction Process

In total, 2 reviewers (GC, BV, PA, GR, and GF; 2 per article) independently extracted relevant data (or informal evidence) using a data extraction form developed specifically for this review and integrated into Covidence. The form was pilot-tested on 2 randomly selected studies and refined accordingly. Data pertaining to the following domains were extracted from the included studies: (1) Study characteristics included information on the first and corresponding authors, publication year, country of origin, aims and hypotheses, study period, design (including details on randomization and blinding), follow-up times, data collection methods, and types of statistical analysis. (2) Participant characteristics included study target population, participant inclusion and exclusion criteria, sex or gender, mean age, and sample sizes at each data collection time point. (3) Intervention characteristics, for which the research team developed a matrix inspired by the template for intervention description and replication 12-item checklist [61] to extract informal evidence (ie, intervention descriptions) from the included studies under the headings name of intervention, purpose, underpinning theory of design elements, treatment approach, type of technology (ie, web or mobile) and software used, delivery format (ie, self-directed, human involvement, or both), provider characteristics (if applicable), intervention duration (ie, length of treatment and number of sessions or modules), material and procedures (ie, tools or activities offered, resources provided, and psychoeducational content), tailoring, and unplanned modifications. (4) Comparator characteristics were details of the control or comparison group or groups, including nature (passive vs active), number of groups or clusters (if applicable), type and length of the intervention (if applicable), and number of participants at each data collection time point. (5) Outcome variables, including the primary outcome variable examined in this systematic review, that is, the mean difference in CU frequency before and after the intervention and between the experimental and control or comparison groups. When possible, we examined continuous variables, including CU frequency means and SDs at the baseline and follow-up time points, and standardized regression coefficients (ie, β coefficients and associated 95% CIs). The secondary outcomes examined included other CU outcome variables (eg, quantity of cannabis used and abstinence) and cannabis-related negative consequences (or problems). Details on outcome variables (ie, definition, data time points, and missing data) and measurements (ie, instruments, measurement units, and scales) were also extracted.

In addition, data on user engagement and use of the digital intervention and study attrition rates (ie, dropouts and loss to follow-up) were extracted. When articles had missing data, we contacted the corresponding authors via email (2 attempts were made over a 2-month period) to obtain missing information. Disagreements over the extracted data were limited and resolved through discussion.

### Data Synthesis Methods

#### Descriptive Synthesis

The characteristics of the included studies, study participants, interventions, and comparators were summarized in narrative and table formats. The template for intervention description and replication 12-item checklist [61] was used to summarize and organize intervention characteristics and assess to what extent the interventions were appropriately described in the included articles. As not all studies had usable data for meta-analysis purposes and because of heterogeneity, we summarized the main findings (ie, intervention effects) of the included studies in narrative and table formats for each outcome of interest in this review.

#### BCT Coding

The BCTs used in the digital interventions were identified from the descriptions of the interventions (ie, experimental groups) provided in the articles as well as any supplementary material and previously published research protocols. A BCT was defined as “an observable, replicable, and irreducible component of an intervention designed to alter or redirect causal processes that regulate behavior” [48]. The target behavior in this review was the cessation or reduction of CU by young adults. BCTs were identified and coded using the BCTTv1 [48,49], a taxonomy of 93 BCTs organized into 16 hierarchical thematic clusters or categories. Applying the BCTTv1 in a systematic review allows for the comparison and synthesis of evidence across studies in a structured manner. This analysis allows for the identification of the explicit mechanisms underlying the reported behavior change induced by interventions, successful or not, and, thus, avoids making implicit assumptions about what works [62].

BCT coding was performed by 2 reviewers independently—BV coded all studies, and GC and GF coded a subset of the studies. All reviewers completed web-based training on the BCTTv1, and GF is an experienced implementation scientist who had used the BCTTv1 in prior work [63-65]. The descriptions of the interventions in the articles were read line by line and analyzed for the clear presence of BCTs using the guidelines developed by Michie et al [48]. For each article, the BCTs identified were documented and categorized using supporting textual evidence. They were coded only once per article regardless of how many times they came up in the text. Disagreements about including a BCT were resolved through discussion. If there was uncertainty about whether a BCT was present, it was coded as absent. Excel (Microsoft Corp) was used to compare the reviewers’ independent BCT coding and generate an overall descriptive synthesis of the BCTs identified. The BCTs were summarized by study and BCT cluster.

#### Statistical Analysis

Meta-analyses were conducted to estimate the size of the effect of the digital interventions for young adult CU on outcomes of interest at the posttreatment and follow-up assessments compared with control or alternative intervention conditions. The outcome variables considered were (1) CU frequency and other CU outcome variables (eg, quantity of cannabis used and abstinence) at baseline and the posttreatment time point or follow-up measured using standardized instruments of self-reported CU (eg, the timeline followback [TLFB] method) [66] and (2) cannabis-related negative consequences measured using standardized instruments (eg, the Marijuana Problems Scale) [67].

Under our systematic review protocol, ≥2 studies were needed for a meta-analysis. On the basis of previous systematic reviews and meta-analyses in the field of digital CU interventions [31,42-45], we expected between-study heterogeneity regarding outcome assessment. To minimize heterogeneity, we chose to pool studies with similar outcomes of interest based on four criteria: (1) definition of outcome (eg, CU frequency, quantity consumed, and abstinence), (2) type of outcome variable (eg, days of CU in the previous 90 days, days high per week in the previous 30 days, and number of CU events in the previous month) and measure (ie, instruments or scales), (3) use of validated instruments, and (4) posttreatment or follow-up time points (eg, 2 weeks or 1 month after the baseline or 3, 6, and 12 months after the baseline).

Only articles that reported sufficient statistics to compute a valid effect size with 95% CIs were included in the meta-analyses. In the case of articles that were not independent (ie, more than one published article reporting data from the same clinical trial), only 1 was included, and it was represented only once in the meta-analysis for a given outcome variable regardless of whether the data used to compute the effect size were extracted from the original paper or a secondary analysis paper. We made sure that the independence of the studies included in the meta-analysis of each outcome was respected. In the case of studies that had more than one comparator, we used the effect size for each comparison between the intervention and control groups.

Meta-analyses were conducted only for mean differences based on the change from baseline in CU frequency at 3 months after the baseline as measured using the number of self-reported days of use in the previous month. As the true value of the estimated effect size for outcome variables might vary across different trials and samples, we used a random-effects model given that the studies retained did not have identical target populations. The random-effects model incorporates between-study variation in the study weights and estimated effect size [68]. In addition, statistical heterogeneity across studies was assessed using *I*^2^, which measures the proportion of heterogeneity to the total observed dispersion; 25% was considered low, 50% was considered moderate, and 75% was considered high [69]. Because only 3 studies were included in the meta-analysis [70-72], publication bias could not be assessed. All analyses were completed using Stata (version 18; StataCorp) [73].

### Risk-of-Bias Assessment

The risk of bias (RoB) of the included RCTs was assessed using the Cochrane RoB 2 tool at the outcome level [74]. Each distinct risk domain (ie, randomization process, deviations from the intended intervention, missing outcome data, measurement of the outcome, and selection of the reported results) was assessed as “low,” “some concerns,” or “high” based on the RoB 2 criteria. In total, 2 reviewers (GC and BV) conducted the assessments independently. Disagreements were discussed, and if not resolved consensually by the 2, the matter was left for a third reviewer (GF) to settle. The assessments were summarized by risk domain and outcome and converted into figures using the RoB visualization tool *robvis* [75].

## Results

### Search Results

**Figure 1:**
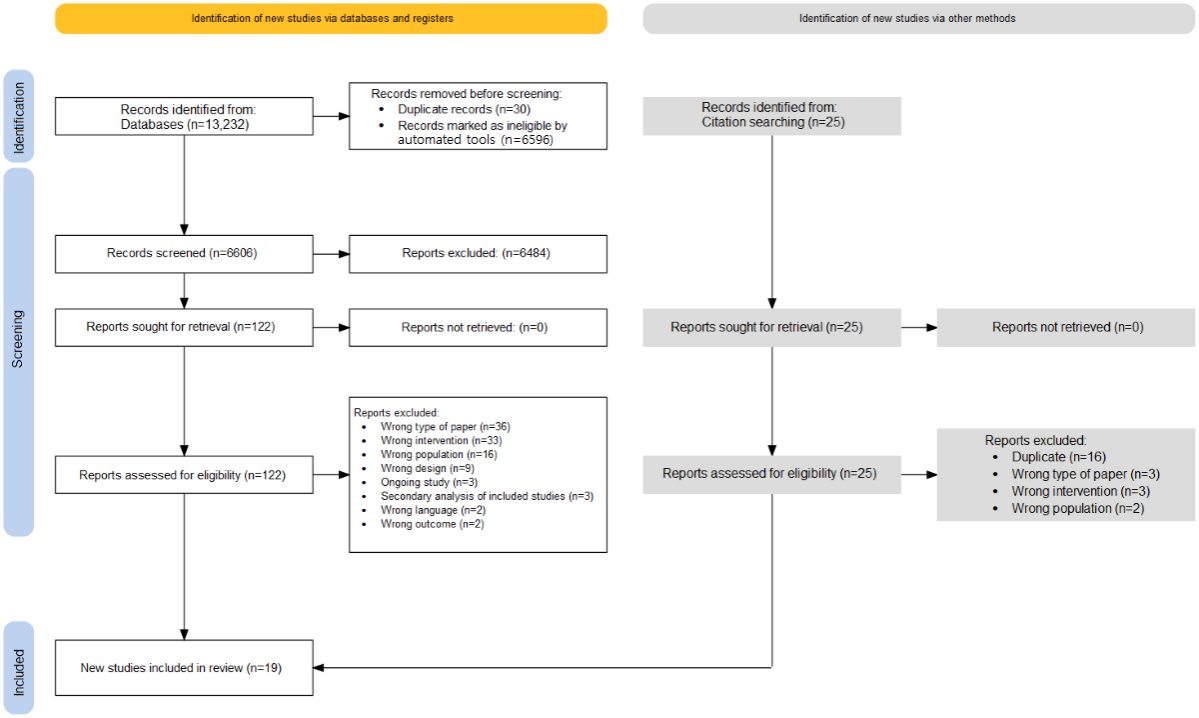
PRISMA (Preferred Reporting Items for Systematic Reviews and Meta-Analyses) study flow diagram.

The database search generated a total of 13,232 citations, of which 7822 (59.11%) were from the initial search on March 18, 2020, and 2805 (21.2%) and 2605 (19.69%) were from the updates on October 13, 2021, and February 13, 2023, respectively. Figure 1 presents the PRISMA study flow diagram [76]. Of the 6606 unique records, 6484 (98.15%) were excluded based on title and abstract screening. Full texts of the remaining 1.85% (122/6606) of the records were examined, as were those of 25 more reports found through hand searching. Of these 147 records, 128 (87.1%) were excluded after 3 rounds of full-text screening. Of these 128 records, 39 (30.5%) were excluded for not being empirical research articles (eg, research protocols). Another 28.1% (36/128) were excluded for not meeting our definition of digital CU intervention. The remaining records were excluded for reasons that occurred with a frequency of ≤14%, including young adults not being the target population and the study not meeting our study design criteria (ie, RCT, cluster RCT, or pilot RCT). Excluded studies and reasons for exclusion are listed in Multimedia Appendix 4. Finally, 19 articles detailing the results of 19 original studies were included.

### Description of Studies

#### Study Characteristics

Multimedia Appendix 5 [70-72,77-92] describes the general characteristics of the 19 included studies. The studies were published between 2010 and 2023, with 58% (11/19) published in 2018 or later. A total of 53% (10/19) of the studies were conducted in the United States [77-86], 11% (2/19) were conducted in Canada [87,88], 11% (2/19) were conducted in Australia [71,89], 11% (2/19) were conducted in Germany [72,90], 11% (2/19) were conducted in Switzerland [70,91], and 5% (1/19) were conducted in Sweden [92]. A total of 79% (15/19) were RCTs [70-72,77,79,81-83,86-92], and 21% (4/19) were pilot RCTs [78,80,84,85].

#### Participant Characteristics

The studies enrolled a total of 6710 participants—3229 (48.1%) in the experimental groups, 3358 (50%) in the control groups, and the remaining 123 (1.8%) from 1 study [82] where participant allocation to the intervention condition was not reported. Baseline sample sizes ranged from 49 [81] to 1292 [72] (mean 352.89, SD 289.50), as shown in Multimedia Appendix 5. Participant mean ages ranged from 18.03 (SD 0.31) [79] to 35.3 (SD 12.6) years [88], and the proportion of participants who identified as female ranged from 24.7% [91] to 84.1% [80].

Of the 19 included studies, 10 (53%) targeted adults aged ≥18 years, of which 7 (70%) studies focused on adults who had engaged in past-month CU [70,71,80,84,85,90,91], 2 (20%) studies included adults who wished to reduce or cease CU [72,89], and 1 (10%) study focused on noncollege adults with a moderate risk associated with CU [88]. Sinadinovic et al [92] targeted young adults aged ≥16 years who had used cannabis at least once a week in the previous 6 months. The remaining 8 studies targeted college or university students (aged ≥17 y) specifically, of which 7 (88%) studies focused solely on students who reported using cannabis [78,79,81-83,86,87] and 1 (12%) study focused solely on students who did not report past-month CU (ie, abstainers) [77].

#### Intervention Characteristics

The 19 included studies assessed nine different digital interventions: (1) 5 (26%) evaluated Marijuana eCHECKUP TO GO (e-TOKE), a commercially available electronic intervention used at colleges throughout the United States and Canada [77,78,81-83]; (2) 2 (11%) examined the internationally known CANreduce program [70,91]; (3) 2 (11%) evaluated the German Quit the Shit program [72,90]; (4) 2 (11%) assessed a social media–delivered, physical activity–focused cannabis intervention [84,85]; (5) 1 (5%) investigated the Swedish *Cannabishjälpen* intervention [92]; (6) 1 (5%) evaluated the Australian Grassessment: Evaluate Your Use of Cannabis website program [89]; (7) 1 (5%) assessed the Canadian *Ma réussite, mon choix* intervention [87]; (8) 1 (5%) examined the Australian Reduce Your Use: How to Break the Cannabis Habit program [71]; and (9) 4 (21%) each evaluated a unique no-name intervention described as a personalized feedback intervention (PFI) [79,80,86,88]. Detailed information regarding the characteristics of all interventions as reported in each included study is provided in Multimedia Appendix 6 [70-72,77-113] and summarized in the following paragraphs.

In several studies (8/19, 42%), the interventions were designed to support cannabis users in reducing or ceasing their consumption [70,72,80,87,89-92]. In 37% (7/19) of the studies, the interventions aimed at reducing both CU and cannabis-related consequences [79,81-85,88]. Other interventions focused on helping college students think carefully about the decision to use cannabis [77,78] and on reducing either cannabis-related problems among undergraduate students [86] or symptoms associated with CU disorder in young adults [71].

In 26% (5/19) of the studies, theory was used to inform intervention design along with a clear rationale for theory use. Of these 5 articles, only 1 (20%) [87] reported using a single theory of behavior change, the theory of planned behavior [114]. A total of 21% (4/19) of the studies selected only constructs of theories (or models) for their intervention design. Of these 4 studies, 2 (50%) evaluated the same intervention [72,90], which focused on principles of self-regulation and self-control theory [93]; 1 (25%) [70] used the concept of adherence-focused guidance enhancement based on the supportive accountability model of guidance [94]; and 1 (25%) [71] reported that intervention design was guided by the concept of self-behavioral management.

The strategies (or approaches) used in the delivery of the digital interventions were discussed in greater detail in 84% (16/19) of the articles [70-72,79-81,83-92]. Many of these articles (9/19, 47%) reported using a combination of approaches based on CBT or motivational interviewing (MI) [70,71,79,83-85,90-92]. PFIs were also often mentioned as an approach to inform intervention delivery [7,71,79,86-88].

More than half (13/19, 68%) of all the digital interventions were asynchronous and based on a self-guided approach without support from a counselor or therapist. The study by Côté et al [87] evaluated the efficacy of a web-based tailored intervention focused on reinforcing a positive attitude toward and a sense of control over cannabis abstinence through psychoeducational messages delivered by a credible character in short video clips and personalized reinforcement messages. Lee et al [79] evaluated a brief, web-based personalized feedback selective intervention based on the PFI approach pioneered by Marlatt et al [95] for alcohol use prevention and on the MI approach described by Miller and Rollnick [96]. Similarly, Rooke et al [71] combined principles of MI and CBT to develop a web-based intervention delivered via web modules, which were informed by previous automated feedback interventions targeting substance use. The study by Copeland et al [89] assessed the short-term effectiveness of Grassessment: Evaluate Your Use of Cannabis, a brief web-based, self-complete intervention based on motivational enhancement therapy that included personalized feedback messages and psychoeducational material. In the studies by Buckner et al [80], Cunningham et al [88], and Walukevich-Dienst et al [86], experimental groups received a brief web-based PFI available via a computer. A total of 16% (3/19) of the studies [77,78,82] applied a program called the Marijuana eCHECKUP TO GO (e-TOKE) for Universities and Colleges, which was presented as a web-based, norm-correcting, brief preventive and intervention education program designed to prompt self-reflection on consequences and consideration of decreasing CU among students. Riggs et al [83] developed and evaluated an adapted version of e-TOKE that provided participants with university-specific personalized feedback and normative information based on protective behavioral strategies for CU [97]. Similarly, Goodness and Palfai [81] tested the efficacy of eCHECKUP TO GO-cannabis, a modified version of e-TOKE combining personalized feedback, norm correction, and a harm and frequency reduction strategy where a “booster” session was provided at 3 months to allow participants to receive repeated exposure to the intervention.

In the remaining 32% (6/19) of the studies, which examined 4 different interventions, the presence of a therapist guide was reported. The intervention evaluated by Sinadinovic et al [92] combined principles of psychoeducation, MI, and CBT organized into 13 web-based modules and a calendar involving therapist guidance, recommendations, and personal feedback. In total, 33% (2/6) of these studies evaluated a social media–delivered intervention with e-coaches that combined principles of MI and CBT and a harm reduction approach for risky CU [84,85]. Schaub et al [91] evaluated the efficacy of CANreduce, a web-based self-help intervention based on both MI and CBT approaches, using automated motivational and feedback emails, chat with a counselor, and web-based psychoeducational modules. Similarly, Baumgartner et al [70] investigated the effectiveness of CANreduce 2.0, a modified version of CANreduce, using semiautomated motivational and adherence-focused guidance-based email feedback with or without a personal online coach. The studies by Tossman et al [72] and Jonas et al [90] used a solution-focused approach and MI to evaluate the effectiveness of the German Quit the Shit web-based program that involves weekly feedback provided by counselors.

In addition to using different intervention strategies or approaches, the interventions were diverse in terms of the duration and frequency of the program (eg, web-based activities, sessions, or modules). Of the 12 articles that provided details in this regard, 2 (17%) on the same intervention described it as a brief 20- to 45-minute web-based program [77,78], 2 (17%) on 2 different interventions reported including 1 or 2 modules per week for a duration of 6 weeks [71,92], and 7 (58%) on 4 different interventions described them as being available over a longer period ranging from 6 weeks to 3 months [70,72,79,84,85,87,90,91].

#### Comparator Types

A total of 42% (8/19) of the studies [72,77-80,85,87,92] used a passive comparator only, namely, a waitlist control group (Multimedia Appendix 5). A total of 26% (5/19) of the studies used an active comparator only where participants were provided with minimal general health feedback regarding recommended guidelines for sleep, exercise, and nutrition [81,82]; strategies for healthy stress management [83]; educational materials about risky CU [88]; or access to a website containing information about cannabis [71]. In another 21% (4/19) of the studies, which used an active comparator, participants received the same digital intervention minus a specific component: a personal web-based coach [70], extended personalized feedback [89], web-based chat counseling [91], or information on risks associated with CU [86]. A total of 21% (4/19) of the studies had more than one control group [70,84,90,91].

### Outcome Variable Assessment and Summary of Main Findings of the Studies

#### Overview

The methodological characteristics and major findings of the included studies (N=19) are presented in Multimedia Appendix 7 [67,70-72,77-92,115-120] and summarized in the following sections for each outcome of interest in this review (ie, CU and cannabis-related consequences). Of the 19 studies, 11 (58%) were reported as efficacy trials [7,77,79,81-83,86-88,91,92], and 8 (42%) were reported as effectiveness trials [70-72,78,84,85,89,90].

Across all the included studies (19/19, 100%), participant attrition rates ranged from 1.6% at 1 month after the baseline [77,78] to 75.1% at the 3-month follow-up [70]. A total of 37% (7/19) of the studies assessed and reported results regarding user engagement [71,78,84,85,90-92] using different types of metrics. In one article on the Marijuana eCHECKUP TO GO (e-TOKE) web-based program [78], the authors briefly reported that participation was confirmed for 98.1% (158/161) of participants in the intervention group. In 11% (2/19) of the studies, which were on a similar social media–delivered intervention [84,85], user engagement was quantified by tallying the number of comments or posts and reactions (eg, likes and hearts) left by participants. In both studies [84,85], the intervention group, which involved a CU-related Facebook page, displayed greater interactions than the control groups, which involved a Facebook page unrelated to CU. One article [84] reported that 80% of participants in the intervention group posted at least once (range 0-60) and 50% posted at least weekly. In the other study [85], the results showed that intervention participants engaged (ie, posting or commenting or clicking reactions) on average 47.9 times each over 8 weeks. In total, 11% (2/19) of the studies [90,91] on 2 different web-based intervention programs, both consisting of web documentation accompanied by chat-based counseling, measured user engagement either by average duration or average number of chat sessions. Finally, 16% (3/19) of the studies [71,91,92], which involved 3 different web-based intervention programs, characterized user engagement by the mean number of web modules completed per participant. Overall, the mean number of web modules completed reported in these articles was quite similar: 3.9 out of 13 [92] and 3.2 [91] and 3.5 [71] out of 6.

#### Assessment of CU

As presented in Multimedia Appendix 7, the included studies differed in terms of how they assessed CU, although all used at least one self-reported measure of frequency. Most studies (16/19, 84%) measured frequency by days of use, including days of use in the preceding week [91] or 2 [80], days of use in the previous 30 [70-72,78,84-86,88-90] or 90 days [79,81,82], and days high per week [83]. Other self-reported measures of CU frequency included (1) number of CU events in the previous month [87,90], (2) cannabis initiation or use in the previous month (ie, yes or no) [77], and (3) days without CU in the previous 7 days [92]. In addition to measuring CU frequency, 42% (8/19) of the studies also assessed CU via self-reported measures of quantity used, including estimated grams consumed in the previous week [92] or 30 days [72,85,90] and the number of standard-sized joints consumed in the previous 7 days [91] or the previous month [70,71,89].

Of the 19 articles included, 10 (53%) [70-72,80,84-86,89,90,92] reported using a validated instrument to measure CU frequency or quantity, including the TLFB instrument [66] (n=9, 90% of the studies) and the Marijuana Use Form (n=1, 10% of the studies); 1 (10%) [79] reported using CU-related questions from an adaptation of the Global Appraisal of Individual Needs–Initial instrument [115]; and 30% (3/10) [81,82,91] reported using a questionnaire accompanied by a calendar or a diary of consumption. The 19 studies also differed with regard to their follow-up time measurements for assessing CU, ranging from 2 weeks after the baseline [80] to 12 months after randomization [90], although 12 (63%) of the studies included a 3-month follow-up assessment [70-72,79,81,82,84,85,88,90-92].

Of all studies assessing and reporting change in CU frequency from baseline to follow-up assessments (19/19, 100%), 47% (9/19) found statistically significant differences between the experimental and control groups [70-72,80,81,83,85,87,91]. Importantly, 67% (6/9) of these studies showed that participants in the experimental groups exhibited greater decreases in CU frequency 3 months following the baseline assessment compared with participants in the control groups [70-72,81,85,91], 22% (2/9) of the studies showed greater decreases in CU frequency at 6 weeks after the baseline assessment [71,83], 22% (2/9) of the studies showed greater decreases in CU frequency at 6 months following the baseline assessment [81,85], 11% (1/9) of the studies showed greater decreases in CU frequency at 2 weeks after the baseline [80], and 11% (1/9) of the studies showed greater decreases in CU frequency at 2 months after treatment [87].

In the study by Baumgartner et al [70], a reduction in CU days was observed in all groups, but the authors reported that the difference was statistically significant only between the intervention group with the service team and the control group (the reduction in the intervention group with social presence was not significant). In the study by Bonar et al [85], the only statistically significant difference between the intervention and control groups at the 3- and 6-month follow-ups involved total days of cannabis vaping in the previous 30 days. Finally, in the study by Buckner et al [80], the intervention group had less CU than the control group 2 weeks after the baseline; however, this was statistically significant only for participants with moderate or high levels of social anxiety.

#### Assessment of Cannabis-Related Negative Consequences

A total of 53% (10/19) of the studies also assessed cannabis-related negative consequences [78-84,86,88,92]. Of these 10 articles, 8 (80%) reported using a validated self-report instrument: 4 (50%) [81,82,86,88] used the 19-item Marijuana Problems Scale [67], 2 (25%) [78,79] used the 18-item Rutgers Marijuana Problem Index [121,122], and 2 (25%) [80,84] used the Brief Marijuana Consequences Questionnaire [116]. Only 10% (1/10) of the studies [92] used a screening tool, the Cannabis Abuse Screening Test [117,118]. None of these 10 studies demonstrated a statistically significant difference between the intervention and control groups. Of note, Walukevich-Dienst et al [86] found that women (but not men) who received an web-based PFI with additional information on CU risks reported significantly fewer cannabis-related problems than did women in the control group at 1 month after the intervention (*B*=−1.941; *P*=.01).

### Descriptive Summary of BCTs Used in Intervention Groups

After the 19 studies included in this review were coded, a total of 184 individual BCTs targeting CU in young adults were identified. Of these 184 BCTs, 133 (72.3%*)* were deemed to be present beyond a reasonable doubt, and 51 (27.7%) were deemed to be present in all probability. Multimedia Appendix 8 [48,70-72,77-92] presents all the BCTs coded for each included study summarized by individual BCT and BCT cluster.

The 184 individual BCTs coded covered 38% (35/93) of the BCTs listed in the BCTTv1 [48]. The number of individual BCTs identified per study ranged from 5 to 19, with two-thirds of the 19 studies (12/19, 63%) using ≤9 BCTs (mean 9.68). As Multimedia Appendix 8 shows, at least one BCT fell into 13 of the 16 possible BCT clusters. The most frequent clusters were *feedback monitoring*, *natural consequences*, *goal planning*, and *comparison of outcomes*.

The most frequently coded BCTs were (1) *feedback on behavior* (BCT 2.2; 17/19, 89% of the studies; eg, “Once a week, participants receive detailed feedback by their counselor on their entries in diary and exercises. Depending on the involvement of each participant, up to seven feedbacks are given” [90]), (2) *social support (unspecified)* (BCT 3.1; 15/19, 79% of the studies; eg, “The website also features [...] blogs from former cannabis users, quick assist links, and weekly automatically generated encouragement emails” [71]), and (3) *pros and cons* (BCT 9.2; 14/19, 74% of the studies; eg, “participants are encouraged to state their personal reasons for and against their cannabis consumption, which they can review at any time, so they may reflect on what they could gain by successfully completing the program” [70]). Other commonly identified BCTs included *social comparison* (BCT 6.2; 12/19, 63% of the studies) and *information about social and environmental consequences* (BCT 5.3; 11/19, 58% of the studies), followed by *problem solving* (BCT 2.1; 10/19, 53% of the studies) and *information about health consequences* (BCT 5.1; 10/19, 53% of the studies).

### RoB Assessment

**Figure 2:**
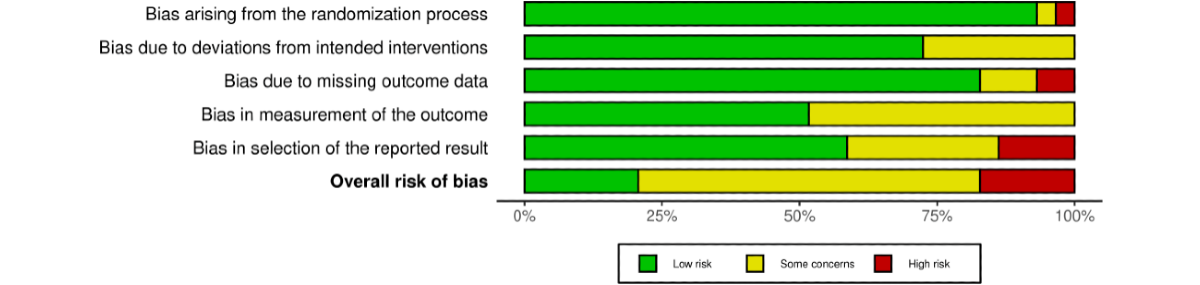
Risk-of-bias (RoB) assessment of the included studies (N=19) summarized by RoB domain.

**Figure 3:**
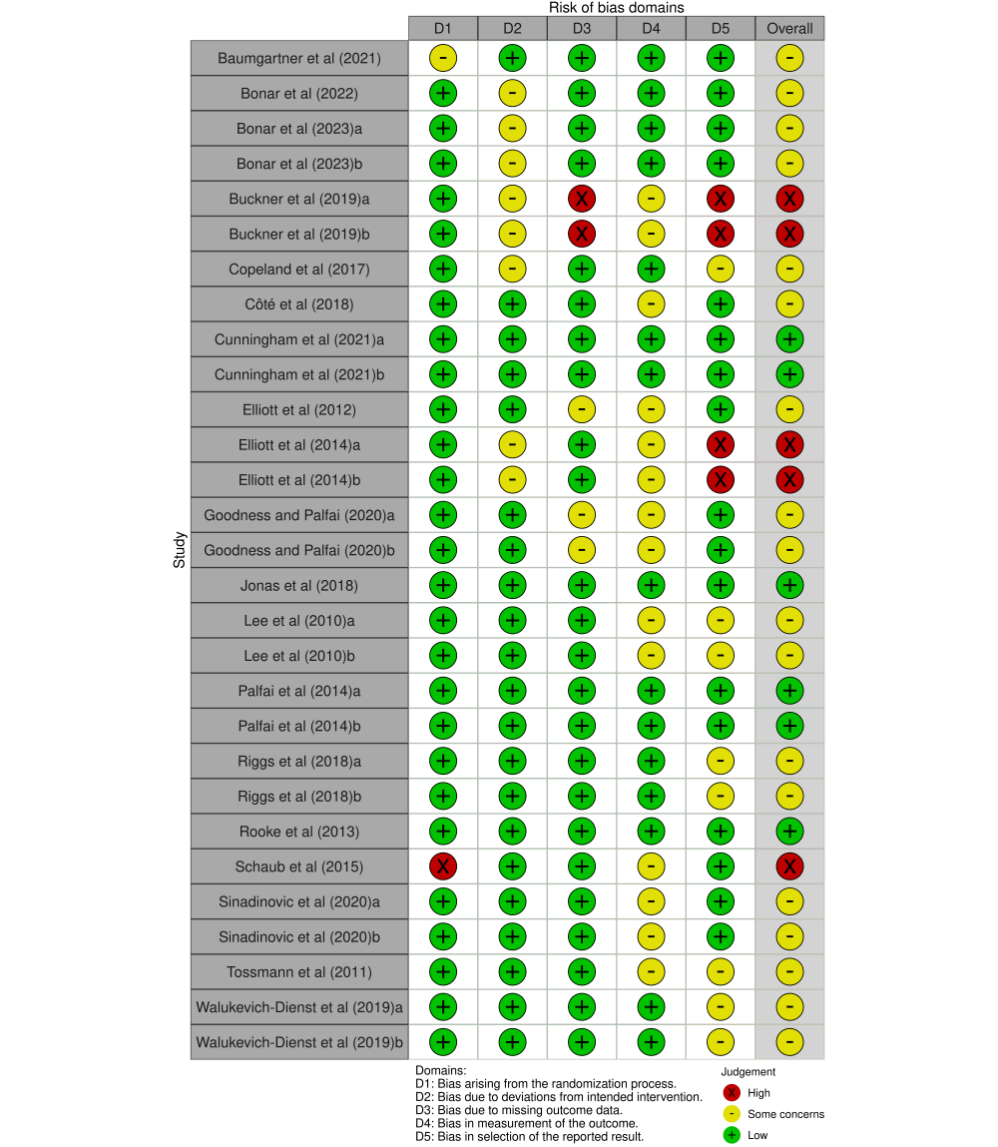
Summary of risk-of-bias (RoB) assessments of each included study (N=19) at the outcome level for each RoB domain.

Figure 2 presents the overall assessment of risk in each domain for all the included studies, whereas Figure 3 [70-72,77-92] summarizes the assessment of each study at the outcome level for each domain in the Cochrane RoB 2 [74].

Figure 2 shows that, of the included studies, 93% (27/29) were rated as having a “low” RoB arising from the randomization process (ie, selection bias) and 83% (24/29) were rated as having a “low” RoB due to missing data (ie, attrition bias). For bias due to deviations from the intended intervention (ie, performance bias), 72% (21/29) were rated as having a “low” risk, and for selective reporting of results, 59% (17/29) were rated as having a “low” risk. In the remaining domain regarding bias in measurement of the outcome (ie, detection bias), 48% (14/29) of the studies were deemed to present “some concerns,” mainly owing to the outcome assessment not being blinded (eg, self-reported outcome measure of CU). Finally, 79% (15/19) of the included studies were deemed to present “some concerns” or were rated as having a “high” RoB at the outcome level (Figure 3 [70-72,77-92]). The RoB assessment for CU and cannabis consequences of each included study is presented in Multimedia Appendix 9 [70-72,77-92].

### Meta-Analysis Results

Due to several missing data points and despite contacting the authors, we were able to carry out only 1 meta-analysis of our primary outcome, CU frequency. Usable data were retrieved from only 16% (3/19) [70-72] of the studies included in this review. These 3 studies provided sufficient information to calculate an effect size, including mean differences based on change-from-baseline measurements and associated 95% CIs (or SE of the mean difference) and sample sizes per intervention and comparison conditions. The reasons for excluding the other 84% (16/19) of the studies included heterogeneity in outcome variables or measurements, inconsistent results, and missing data (Multimedia Appendix 10 [77-92]).

**Figure 4:**
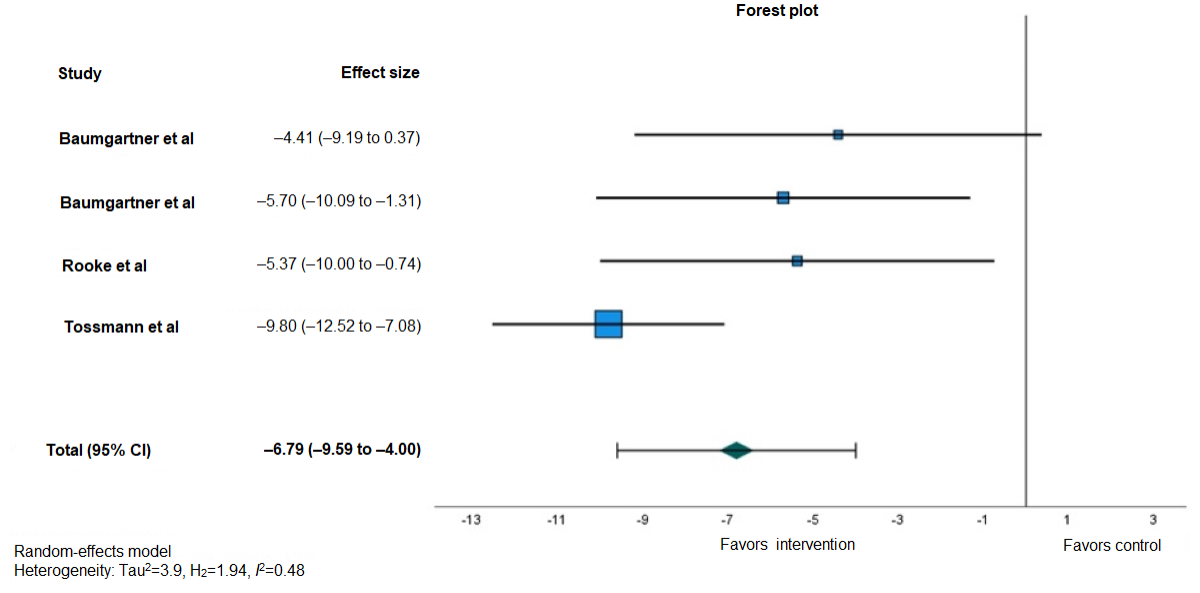
Forest plot displaying random-effects meta-analysis of the effect of digital interventions for young adult cannabis users on cannabis use frequency at 3 months after the baseline relative to a comparison condition measured using the number of self-reported days of use in the previous month through the timeline followback method.

Figure 4 [70-72] illustrates the mean differences and associated 95% CIs of 3 unique RCTs [70-72] that provided sufficient information to allow for the measurement of CU frequency at 3 months after the baseline relative to a comparison condition in terms of the number of self-reported days of use in the previous month using the TLFB method. Overall, the synthesized effect of digital interventions for young adult cannabis users on CU frequency, as measured using days of use in the previous month, was −6.79 (95% CI −9.59 to −4.00). This suggests that digital CU interventions had a statistically significant effect (*P*<.001) on reducing CU frequency at the 3-month follow-up compared with the control conditions (both passive and active controls). The results of the meta-analysis also showed low between-study heterogeneity (*I*^2^=48.3%; *P*=.12) across the 3 included studies.

The samples of the 3 studies included in the meta-analysis varied in size from 225 to 1292 participants (mean 697.33, SD 444.11), and the mean age ranged from 24.7 to 31.88 years (mean 26.38, SD 3.58 years). These studies involved 3 different digital interventions and used different design approaches to assess intervention effectiveness. One study assessed the effectiveness of a web-based counseling program (ie, Quit the Shit) against a waitlist control [72], another examined the effectiveness of a fully self-guided web-based treatment program for CU and related problems (ie, Reduce Your Use: How to Break the Cannabis Habit) against a control condition website consisting of basic educational information on cannabis [71], and the third used a 3-arm RCT design to investigate whether the effectiveness of a minimally guided internet-based self-help intervention (ie, CANreduce 2.0) might be enhanced by implementing adherence-focused guidance and emphasizing the social presence factor of a personal e-coach [70].

## Discussion

### Summary of Principal Findings

The primary aim of this systematic review was to evaluate the effectiveness of digital interventions in addressing CU among community-living young adults. We included 19 randomized controlled studies representing 9 unique digital interventions aimed at preventing, reducing, or ceasing CU and evaluated the effects of 3 different digital interventions on CU. In summary, the 3 digital interventions included in the meta-analysis proved superior to control conditions in reducing the number of days of CU in the previous month at the 3-month follow-up.

Our findings are consistent with those of 2 previous meta-analyses by Olmos et al [43] and Tait et al [44] and with the findings of a recently published umbrella review of systematic reviews and meta-analyses of RCTs [123], all of which revealed a positive effect of internet- and computer-based interventions on CU. However, a recent systematic review and meta-analysis by Beneria et al [45] found that web-based CU interventions did not significantly reduce CU. Beneria et al [45] included studies with different intervention programs that targeted diverse population groups (both adolescents and young adults) and use of more than one substance (eg, alcohol and cannabis). In our systematic review, a more conservative approach was taken—we focused specifically on young adults and considered interventions targeting CU only. Although our results indicate that digital interventions hold great promise in terms of effectiveness, an important question that remains unresolved is whether there is an optimal exposure dose in terms of both duration and frequency that might be more effective. Among the studies included in this systematic review, interventions varied considerably in terms of the number of psychoeducational modules offered (from 2 to 13), time spent reviewing the material, and duration (from a single session to a 12-week spread period). Our results suggest that an intervention duration of at least 6 weeks yields better results.

Another important finding of this review is that, although almost half (9/19, 47%) of the included studies observed an intervention effect on CU frequency, none reported a statistically significant improvement in cannabis-related negative consequences, which may be considered a more distal indicator. More than half (10/19, 53%) of the included studies investigated this outcome. It seems normal to expect to find an effect on CU frequency given that reducing CU is often the primary objective of interventions and because the motivation of users’ is generally focused on changing consumption behavior. It is plausible to think that the change in behavior at the consumption level must be maintained over time before an effect on cannabis-related negative consequences can be observed. However, our results showed that, in all the included studies, cannabis-related negative consequences and change in behavior (CU frequency) were measured at the same time point, namely, 3 months after the baseline. Moreover, Grigsby et al [124] conducted a scoping review of risk and protective factors for CU and suggested that interventions to reduce negative CU consequences should prioritize multilevel methods or strategies “to attenuate the cumulative risk from a combination of psychological, contextual, and social influences.”

A secondary objective of this systematic review was to describe the active ingredients used in digital interventions for CU among young adults. The vast majority of the interventions were based on either a theory or an intervention approach derived from theories such as CBT, MI, and personalized feedback. From these theories and approaches stem behavior change strategies or techniques, commonly known as BCTs. *Feedback on behavior*, included in the *feedback monitoring* BCT cluster, was the most common BCT used in the included studies. This specific BCT appears to be a core strategy in behavior change interventions [125,126]. In their systematic review of remotely delivered alcohol or substance misuse interventions for adults, Howlett et al [53] found that *feedback on behavior*, *problem solving*, and *goal setting* were the most frequently used BCTs in the included studies. In addition, this research group noted that the most promising BCTs for alcohol misuse were *avoidance/reducing exposure to cues for behavior*, *pros and cons*, and *self-monitoring of behavior,* whereas 2 very promising strategies for substance misuse in general were *problem solving* and *self-monitoring of behavior*. In our systematic review, in addition to *feedback on behavior*, the 6 most frequently used BCTs in the included studies were *social support*, *pros and cons*, *social comparison*, *problem solving*, *information about social and environmental consequences*, and *information about health consequences*. Although *pros and cons* and *problem solving* were present in all 3 studies of digital interventions included in our meta-analysis, *avoidance/reducing exposure to cues for behavior* was reported in only 5% (1/19) of the articles, and *feedback on behavior* was more frequently used than *self-monitoring of behavior.* However, it should be noted that the review by Howlett et al [53] examined digital interventions for participants with alcohol or substance misuse problems, whereas in this review, we focused on interventions that targeted CU from a harm reduction perspective. In this light, *avoidance/reducing exposure to cues for behavior* may be a BCT better suited to populations with substance misuse problems. Lending support to this, a meta-regression by Garnett et al [127] and a Cochrane systematic review by Kaner et al [128] both found interventions that used *behavior substitution* and *credible source* to be associated with greater reduction in excessive alcohol consumption compared with interventions that used other BCTs.

Beyond the number and types of BCTs used, reflecting on the extent to which each BCT in a given intervention suits (or does not suit) the targeted determinants (ie, behavioral and environmental causes) is crucial for planning intervention programs [26]. It is important when designing digital CU interventions not merely to pick a combination of BCTs that have been associated with effectiveness. Rather, the active ingredients must fit the determinants that the interventionists seek to influence. For example, *action planning* would be more relevant as a BCT for young adults highly motivated and ready to take action on their CU than would *pros and cons*, which aims instead to bolster motivation. Given that more than half of all digital interventions are asynchronous and based on a self-guided approach and do not offer counselor or therapist support, a great deal of motivation is required to engage in intervention and behavior change. Therefore, it is essential that developers consider the needs and characteristics of the targeted population to tailor intervention strategies (ie, BCTs) for successful behavior change (eg, tailored to the participant’s stage of change). In most of the digital interventions included in this systematic review, personalization was achieved through feedback messages about CU regarding descriptive norms, motives, risks and consequences, and costs, among other things.

Despite the high number of recent studies conducted in the field of digital CU interventions, most of the included articles in our review (17/19, 89%) reported on the development and evaluation of web-based intervention programs. A new generation of health intervention modalities such as mobile apps and social media has drawn the attention of researchers in the past decade and is currently being evaluated. In this regard, the results from a recently published scoping review [129], which included 5 studies of mobile apps for nonmedical CU, suggested that these novel modes of intervention delivery demonstrated adequate feasibility and acceptability. Nevertheless, the internet remains a powerful and convenient medium for reaching young adults with digital interventions intended to support safe CU behaviors [123,130].

### Quality of Evidence

The GRADE (Grading of Recommendations Assessment, Development, and Evaluation) approach [131-133] was used to assess the quality of the evidence reviewed. It was deemed to be moderate for the primary outcome of this review, that is, CU frequency in terms of days of use in the previous month (see the summary of evidence in Multimedia Appendix 11 [70,72]). The direction of evidence was broadly consistent—in all 3 RCT studies [70-72] included in the meta-analysis, participants who received digital CU interventions reduced their consumption compared with those who received no or minimal interventions. The 3 RCTs were similar in that they all involved a web-based, multicomponent intervention program aimed at reducing or ceasing CU. However, the interventions did differ or vary in terms of several characteristics, including the strategies used, content, frequency, and duration. Given the small number of studies included in the meta-analysis, we could not conclude with certainty which intervention components, if any, contributed to the effect estimate observed.

Although inconsistency, indirectness, and imprecision were not major issues in the body of evidence, we downgraded the evidence from high to moderate quality on account of RoB assessments at the outcome level. The 3 RCT studies included in the meta-analysis were rated as having “some concerns” of RoB, mainly due to lack of blinding, which significantly reduced our certainty relative to subjective outcomes (ie, self-reported measures of CU frequency). A positive feature of these digital intervention trials is that most procedures are fully automated, and so there was typically a low RoB regarding randomization procedures, allocation to different conditions, and intervention delivery. It is impossible to blind participants to these types of behavior change interventions, and although some researchers have made attempts to counter the impact of this risk, performance bias is an inescapable issue in RCT studies of this kind. Blinding of intervention providers was not an issue in the 3 RCTs included in the meta-analysis because outcome data collection was automated. However, this same automated procedure made it very difficult to ensure follow‐up. Consequently, attrition was another source of bias in these RCT studies [70-72]. The participants lost to follow-up likely stopped using the intervention. However, there is no way of determining whether these people would have benefited more or less than the completers if they had seen the trial through.

The 3 RCTs included in the meta-analysis relied on subjective self-reported measures of CU at baseline and follow‐up, which are subject to recall and social desirability bias. However, all 3 studies used a well-validated instrument of measurement to determine frequency of CU, the TLFB [66]. This is a widely used, subjective self-report tool for measuring frequency (or quantity) of substance use (or abstinence). It is considered a reliable measure of CU [134,135]. Finally, it should be pointed out that any potential bias related to self‐reported CU frequency would have affected both the intervention and control groups (particularly in cases in which control groups received cannabis‐related information), and thus, it was unlikely to account for differential intervention effects. Moreover, we found RoB due to selective reporting in some studies owing mainly to the absence of any reference to a protocol. Ultimately, these limitations may have biased the results of the meta-analysis. Consequently, future research is likely to further undermine our confidence in the effect estimate we observed and report considerably different estimates.

### Strengths and Limitations

Our systematic review and meta-analysis has a number of strengths: (1) we included only randomized controlled studies to ensure that the included studies possessed a rigorous research design, (2) we focused specifically on cannabis (rather than combining multiple substances), (3) we assessed the effectiveness of 3 different digital interventions on CU frequency among community-living young adults, and (4) we performed an exhaustive synthesis and comparison of the BCTs used in the 9 digital interventions examined in the 19 studies included in our review based on the BCTTv1.

Admittedly, this systematic review and meta-analysis has limitations that should be recognized. First, although we searched a range of bibliographic databases, the review was limited to articles published in peer-reviewed journals in English or French. This may have introduced publication bias given that articles reporting positive effects are more likely to be published than those with negative or equivocal results. Consequently, the studies included in this review may have overrepresented the statistically significant effects of digital CU interventions.

Second, only a small number of studies were included in the meta-analyses because many studies did not provide adequate statistical information for calculating and synthesizing effect sizes, although significant efforts were made to contact the authors in case of missing data. Because of the small sample size used in the meta-analysis, the effect size estimates may not be highly reflective of the true effects of digital interventions on CU frequency among young adults. Furthermore, synthesizing findings across studies that evaluated different modalities of web-based intervention programs (eg, fully self-guided vs with therapist guidance) and types of intervention approaches (eg, CBT, MI, and personalized feedback) may have introduced bias in the meta-analytical results due to the heterogeneity of the included studies, although heterogeneity was controlled for using a random-effects model and our results indicated low between-study heterogeneity.

Third, we took various measures to ensure that BCT coding was carried out rigorously throughout the data extraction and analysis procedures: (1) all coders received training on how to use the BCTTv1; (2) all the included articles were read line by line so that coders became familiar with intervention descriptions before initiating BCT coding; (3) the intervention description of each included article was double coded after a pilot calibration exercise with all coders, and any disagreements regarding the presence or absence of a BCT were discussed and resolved with a third party; and (4) we contacted the article authors when necessary and possible for further details on the BCTs they used. However, incomplete reporting of intervention content is a recognized issue [136], which may have resulted in our coding BCTs incorrectly as present or absent. Reliably specifying the BCTs used in interventions allows their active ingredients to be identified, their evidence to be synthesized, and interventions to be replicated, thereby providing tangible guidance to programmers and researchers to develop more effective interventions.

Finally, although this review identified the BCTs used in digital interventions, our approach did not allow us to draw conclusions regarding their effectiveness. Coding BCTs simply as present or absent does not consider the frequency, intensity, and quality with which they were delivered. For example, it is unclear how many individuals should self‐monitor their CU. In addition, the quality of BCT implementation may be critical in digital interventions where different graphics and interface designs and the usability of the BCTs used can have considerable influence on the level of user engagement [137]. In the future, it may be necessary to develop new methods to evaluate the dosage of individual BCTs in digital health interventions and characterize their implementation quality to assess their effectiveness [128,138]. Despite its limitations, this review suggests that digital interventions represent a promising avenue for preventing, reducing, or ceasing CU among community-living young adults.

### Conclusions

The results of this systematic review and meta-analysis lend support to the promise of digital interventions as an effective means of reducing recreational CU frequency among young adults. Despite the advent and popularity of smartphones, web-based interventions remain the most common mode of delivery for digital interventions. The active ingredients of digital interventions are varied and encompass a number of clusters of the BCTTv1, but a significant number of BCTs remain underused. Additional research is needed to further investigate the effectiveness of these interventions on CU and key outcomes at later time points. Finally, a detailed assessment of user engagement with digital interventions for CU and understanding which intervention components are the most effective remain important research gaps.

## Appendix

Multimedia Appendix 1 PRISMA (Preferred Reporting Items for Systematic Reviews and Meta-Analyses) checklist.

Multimedia Appendix 2 Detailed search strategies for each database.

Multimedia Appendix 3 Population, intervention, comparison, outcome, and study design strategy.

Multimedia Appendix 4 Excluded studies and reasons for exclusion.

Multimedia Appendix 5 Study and participant characteristics.

Multimedia Appendix 6 Description of intervention characteristics in the included articles.

Multimedia Appendix 7 Summary of methodological characteristics and major findings of the included studies categorized by intervention name.

Multimedia Appendix 8 Behavior change techniques (BCTs) coded in each included study summarized by individual BCT and BCT cluster.

Multimedia Appendix 9 Risk-of-bias assessment of each included study for cannabis use and cannabis consequences.

Multimedia Appendix 10 Excluded studies and reasons for exclusion from the meta-analysis.

Multimedia Appendix 11 Summary of evidence according to the Grading of Recommendations Assessment, Development, and Evaluation tool.

## Abbreviations

BCT: behavior change technique
BCTTv1: Behavior Change Technique Taxonomy version 1
CBT: cognitive behavioral therapy
CU: cannabis use
GRADE: Grading of Recommendations Assessment, Development, and Evaluation
MI: motivational interviewing
PICO: population, intervention, comparator, and outcome
PFI: personalized feedback intervention
PRISMA: Preferred Reporting Items for Systematic Reviews and Meta-Analyses
RCT: randomized controlled trial
RoB: risk of bias
TLFB: Timeline Followback

## Data Availability

The data sets generated and analyzed during this study are available from the corresponding author on reasonable request.
