# Supplementary material for: Digital Interventions for Recreational Cannabis Use Among Young Adults: Systematic Review, Meta-Analysis, and Behavior Change Technique Analysis of Randomized Controlled Studies
Source: J Med Internet Res. 2024 Apr 17;26:e55031. doi: 10.2196/55031 (PMC11063887; doi:10.2196/55031)
Supplement: Multimedia Appendix 2 [file jmir_v26i1e55031_app2.pdf]

LITERATURE SEARCH REPORT

**Authors:**	Côté, José; Chicoine, Gabrielle; Vinette, Billy; Auger, Patricia; Rouleau, Geneviève; Fontaine, Guillaume; Jutras-Aswad, Didier.
**Review title:**	Digital interventions for nonmedical cannabis use among young adults: a systematic review, meta-analysis, and behavior change technique analysis
**Date:**	February 13, 2023

This report is based on the PRISMA-S recommendations for reporting systematic reviews[1].

Contents

1. Methodology 2
2. Databases and search strategies 3
   - 2.1. CINAHL Complete (Ebsco) 3
   - 2.2. Cochrane Database of Systematic Reviews and Central Register of Controlled Trials (Ovid) 4
   - 2.3. Embase (Ovid) <1974 to 2023 February 1[illegible]> 5
   - 2.4. Ovid MEDLINE(R) ALL <1946 to February 10, 2023> 6
   - 2.5. PsycINFO (Ovid) <1806 to January Week 5 2023> 7
   - 2.6. PubMed <Oct 6 to Oct 13> 8
3. Results of the search strategy update (February 13, 2023), for each database 9

---

[1] Rethlefsen ML, Kirtley S, Waffenschmidt S, Ayala AP, Moher D, Page MJ, et al. PRISMA-S: an extension to the PRISMA Statement for Reporting Literature Searches in Systematic Reviews. Syst Rev. 2021;10(1):39. DOI: https://dx.doi.org/10.1186/s13643-020-01542-z.

1. Methodology

Information sources and methods

The following electronic databases were searched: MEDLINE (via Ovid; via PubMed for the last 7 days only); Embase (via Ovid); CINAHL Complete (via Ebsco), Cochrane Database of Systematic reviews (via Ovid), Cochrane Central Register of Controlled Trials (via Ovid) and PsycInfo (via Ovid).

Reference lists of included articles were manually screened to identify additional studies.

Search strategies

Search strategies were designed by a librarian (BN).

The comprehensive literature search was initially run on March 18, 2020 and then rerun on October 13th, 2021 and then on February 13th, 2023.

The Medline search strategy was peer reviewed by another senior information specialist prior to execution and then applied to all databases, with modifications to search terms as necessary. No language limits were applied.

Managing records

A total of 7822 citations were retrieved from the 7 databases initially, and 2805 and 2605 for the updates in 2021 and 2023, respectively.

Duplicates were removed in EndNote by the librarian (BN).

After duplicate removal, 4204 remained to be screened on March 18, 2020 and 1316 for the update in 2021 and 1086 in 2023.

2. Databases and search strategies

2.1. CINAHL Complete (Ebsco)

S1	(MH "Cannabis")	11,550
S2	TI (Cannabi* or Marihuana* or Marijuana* or Hemp or Hemps or Ganja or Ganjas or Hashish or Hashishs or Bhang or Bhangs or Smoking Blunt*) OR AB (Cannabi* or Marihuana* or Marijuana* or Hemp or Hemps or Ganja or Ganjas or Hashish or Hashishs or Bhang or Bhangs or Smoking Blunt*)	22,708
S3	TI (weed* N3 (smok* or use* or using or abus* or dependen* or disorder*)) OR AB (weed* N3 (smok* or use* or using or abus* or dependen* or disorder*))	55
S4	S1 OR S2 OR S3	25,263
S5	(MH "Internet")	54,646
S6	(MH "Cellular Phone") OR (MH "Text Messaging") OR (MH "Smartphone")	9,940
S7	(MH "World Wide Web")	78,811
S8	(MH "Telehealth") OR (MH "Telemedicine")	28,742
S9	(MH "Mobile Applications")	11,814
S10	(MH "Microcomputers+")	11,944
S11	(MH "Computer Simulation") OR (MH "Augmented Reality") OR (MH "Virtual Reality")	26,485
S12	(MH "Online Services")	4,911
S13	(MH "Online Social Networking")	630
S14	(MH "Social Media") OR (MH "Facebook") OR (MH "Twitter")	21,931
S15	TI (Web or website* or internet or echeckup or smartphone* or ((ios or android or smart or portable or cell) N2 (device* or phone* or system* or platform* or application*)) or mobile or online or on-line or (chat N2 (live or based or messenger* or group* or interface or function* or forum* or transcript* or session* or log or logs or counsel* or bot or bots or support* or room or rooms)) or computer* or mhealth or m-health or ehealth or e-health or app or apps or digital or SMS or messaging or Short Message Service* or texting or ((virtual or augmented) N2 realit*) or Serious gam* or telemedicine or telehealth* or (tele N2 (medicine or health*)) or blog or blogs or blogging or social media* or (wearable N2 device*) or ((electronic or digital) N3 (communicat* or learn* or game* or gaming))) OR AB (Web or website* or internet or echeckup or smartphone* or ((ios or android or smart or portable or cell) N2 (device* or phone* or system* or platform* or application*)) or mobile or online or on-line or (chat N2 (live or based or messenger* or group* or interface or function* or forum* or transcript* or session* or log or logs or counsel* or bot or bots or support* or room or rooms)) or computer* or mhealth or m-health or ehealth or e-health or app or apps or digital or SMS or messaging or Short Message Service* or texting or ((virtual or augmented) N2 realit*) or Serious gam* or telemedicine or telehealth* or (tele N2 (medicine or health*)) or blog or blogs or blogging or social media* or (wearable N2 device*) or ((electronic or digital) N3 (communicat* or learn* or game* or gaming)))	502,381
S16	S5 OR S6 OR S7 OR S8 OR S9 OR S10 OR S11 OR S12 OR S13 OR S14 OR S15	621,683
S17	S4 AND S16	2,218
S18	TI (newborn* or new-born* or neonat* or neo-nat* or infan* or child* or adolesc* or paediatr* or pediatr* or baby* or babies* or toddler* or kid or kids or boy* or girl* or juvenile* or teen* or youth* or pubescen* or preadolesc* or prepubesc* or preteen or tween)	687,884
S19	SO (pediatr* or paediatr*)	211,156
S20	S18 OR S19	770,308
S21	S17 NOT S20	1,892
S22	(MH "Students") OR (MH "Students, College")	48,275
S23	(MH "Students, Graduate+")	4,927
S24	(MH "Students, Health Occupations+")	83,286
S25	TI (young N2 (adult* or people)) OR AB (young N2 (adult* or people))	70,293

S26	TI ((college* or undergraduate or postgraduate or university) N2 (age* or participant* or student*)) OR AB ((college* or undergraduate or postgraduate or university) N2 (age* or participant* or student*))	45,458
S27	TI ("18 years" or "19 years" or "20 years" or "21 years" or "22 years" or "23 years" or "24 years" or "25 years" or "26 years" or "27 years" or "28 years" or "29 years" or "30 years" or "31 years" or "32 years" or "33 years" or "34 years" or "35 years") OR AB ("18 years" or "19 years" or "20 years" or "21 years" or "22 years" or "23 years" or "24 years" or "25 years" or "26 years" or "27 years" or "28 years" or "29 years" or "30 years" or "31 years" or "32 years" or "33 years" or "34 years" or "35 years")	106,821
S28	S22 OR S23 OR S24 OR S25 OR S26 OR S27	314,161
S29	S17 AND S28	502
S30	S21 OR S29	1,977
S31	EM 202110* OR EM 202111* OR EM 202112* OR EM 2022* OR EM 2023*	530,653
S32	S30 AND S31	349

2.2. Cochrane Database of Systematic Reviews and Central Register of Controlled Trials (Ovid)

Cochrane Central Register of Controlled Trials January 2023
Cochrane Database of Systematic Reviews 2005 to February 8, 2023

1	(Cannabi* or Marihuana* or Marijuana* or Hemp or Hemps or Ganja or Ganjas or Hashish or Hashishs or Bhang or Bhangs or Smoking Blunt*).ti,ab.	4964
2	(weed* adj3 (smok* or use* or using or abus* or dependen* or disorder*)).ti,ab.	8
3	1 or 2	4972
4	(Web or website* or internet or echeckup or smartphone* or ((ios or android or smart or portable or cell) adj2 (device* or phone* or system* or platform* or application*)) or mobile or online or on-line or (chat adj2 (live or based or messenger* or group* or interface or function* or forum* or transcript* or session* or log or logs or counsel* or bot or bots or support* or room or rooms)) or computer* or mhealth or m-health or ehealth or e-health or app or apps or digital or SMS or messaging or Short Message Service* or texting or ((virtual or augmented) adj2 realit*) or Serious gam* or telemedicine or telehealth* or (tele adj2 (medicine or health*)) or blog or blogs or blogging or social media* or (wearable adj2 device*) or ((electronic or digital) adj3 (communicat* or learn* or game* or gaming))).ti,ab.	113077
5	3 and 4	588
6	(newborn* or new-born* or neonat* or neo-nat* or infan* or child* or adolesc* or paediatr* or pediatr* or baby* or babies* or toddler* or kid or kids or boy* or girl* or juvenile* or youth or teen* or pubescen* or preadolesc* or prepubesc* or preteen or tween).ti.	154041
7	5 not 6	490
8	("20220131" or "20220207" or "20220214" or "20220221" or "20220228" or "20220307" or "20220314" or "20220321" or "20220328" or "20220404" or "20220411" or "20220418" or "20220425" or "20220502" or "20220509" or "20220516" or "20220523" or "20220531" or "20220606" or "20220613" or "20220620" or "20220627" or "20220704" or "20220711" or "20220718" or "20220725" or "202208" or "20220801" or "20220808" or "20220815" or "20220822" or "20220829" or "202209" or "20220905" or "20220912" or "20220919" or "20220926" or "202210" or "20221003" or "20221010" or "20221017" or "20221024" or "20221031" or "202211" or "20221107" or "20221114" or "20221121" or "20221128" or "202212" or "20221205" or "20221212" or "20221219" or "20221226" or "202301" or "20230102" or "20230109" or "20230116" or "20230123" or "20230130" or "20230206" or "20211011" or "20211018" or "20211025" or "20211101" or "20211108" or "20211115" or "20211122" or "20211129" or "20211206" or	401436

	"20211213" or "20211220" or "20211227" or "20220103" or "20220110" or "20220117" or "20220124").up.	
9	7 and 8	166
10	("2021" or "2022" or "2023").yr.	214793
11	9 and 10	69

2.3. Embase (Ovid) <1974 to 2023 February 10>

1	cannabis/	42692
2	exp "cannabis use"/	17130
3	cannabis addiction/	11438
4	(Cannabi* or Marihuana* or Marijuana* or Hemp or Hemps or Ganja or Ganjas or Hashish or Hashishs or Bhang or Bhangs or Smoking Blunt*).tw,kw.	78477
5	(weed* adj3 (smok* or use* or using or abus* or dependen* or disorder*)).tw,kw.	777
6	1 or 2 or 3 or 4 or 5	93178
7	internet/	120526
8	exp mobile phone/	44398
9	exp mobile application/	22869
10	computer/	84114
11	exp personal computer/	13101
12	telemedicine/	41574
13	online system/	29810
14	text messaging/	7177
15	personal digital assistant/	1755
16	medical informatics/	22687
17	virtual reality/	24536
18	(Web or website* or internet or echeckup or smartphone* or ((ios or android or smart or portable or cell) adj2 (device* or phone* or system* or platform* or application*)) or mobile or online or on-line or text messag* or texting or (chat adj2 (live or based or messenger* or group* or interface or function* or forum* or transcript* or session* or log or logs or counsel* or bot or bots or support* or room or rooms)) or computer* or mhealth or m-health or ehealth or e-health or app or apps or digital or SMS or messaging or Short Message Service* or texting or ((virtual or augmented) adj2 realit*) or Serious gam* or telemedicine or telehealth* or (tele adj2 (medicine or health*)) or blog or blogs or blogging or social media* or (wearable adj2 device*) or ((electronic or digital) adj3 (communicat* or learn* or game* or gaming))).tw,kw.	1521658
19	or/7-18	1630394
20	6 and 19	5746
21	(exp child/ or exp adolescent/) not exp adult/	2443694
22	(newborn* or new-born* or neonat* or neo-nat* or infan* or child* or adolesc* or paediatr* or pediatr* or baby* or babies* or toddler* or kid or kids or boy* or girl* or juvenile* or teen* or youth* or pubescen* or preadolesc* or prepubesc* or preteen or tween).ti.	2013280
23	(pediatr* or paediatr*).jx.	774514
24	21 or 22 or 23	3235487
25	20 not 24	4834
26	student/ or college student/ or postgraduate student/ or undergraduate student/ or university student/	167594
27	(young adj2 (adult or people)).tw,kw.	99270
28	((college* or undergraduate or postgraduate or university) adj2 (age* or participant* or student*)).tw,kw.	84054

29	("18 years" or "19 years" or "20 years" or "21 years" or "22 years" or "23 years" or "24 years" or "25 years" or "26 years" or "27 years" or "28 years" or "29 years" or "30 years" or "31 years" or "32 years" or "33 years" or "34 years" or "35 years").tw,kw.	769852
30	or/26-29	1038519
31	20 and 30	1141
32	25 or 31	5119
33	remove duplicates from 32	4895
34	("20211013" or "20211014" or "20211015" or "20211016" or "20211017" or "20211018" or "20211019" or 2021102* or 2021103* or 202111* or 202112* or 2022* or 2023*).dc,dd.	2857942
35	33 and 34	1068

2.4. Ovid MEDLINE(R) ALL <1946 to February 10, 2023>

Medline search strategy peer reviewed by a second librarian.

1	Cannabis/	13129
2	exp "Marijuana Use"/	6956
3	Marijuana abuse/	6987
4	(Cannabi* or Marihuana* or Marijuana* or Hemp or Hemps or Ganja or Ganjas or Hashish or Hashishs or Bhang or Bhangs or Smoking Blunt*).tw,kf.	59014
5	(weed* adj3 (smok* or use* or using or abus* or dependen* or disorder*)).tw,kf.	722
6	or/1-5	63043
7	Internet/	80634
8	Internet-Based Intervention/	1076
9	Smartphone/	8654
10	Mobile Applications/	10994
11	Computers/	53096
12	Telemedicine/	36166
13	Online systems/	8545
14	Text messaging/	4379
15	Cell phone/	9897
16	Computers handheld/	4034
17	Medical Informatics Applications/	2550
18	Virtual reality/	5141
19	Augmented reality/	1043
20	(Web or website* or internet or echeckup or smartphone* or ((ios or android or smart or portable or cell) adj2 (device* or phone* or system* or platform* or application*)) or mobile or online or on-line or (chat adj2 (live or based or messenger* or group* or interface or function* or forum* or transcript* or session* or log or logs or counsel* or bot or bots or support* or room or rooms)) or computer* or mhealth or m-health or ehealth or e-health or app or apps or digital or SMS or messaging or Short Message Service* or texting or ((virtual or augmented) adj2 realit*) or Serious gam* or telemedicine or telehealth* or (tele adj2 (medicine or health*)) or blog or blogs or blogging or social media* or (wearable adj2 device*) or ((electronic or digital) adj3 (communicat* or learn* or game* or gaming))).tw,kf.	1197077
21	or/7-20	1257184
22	6 and 21	3591
23	(exp child/ or exp infant/ or adolescent/) not exp adult/	2109159
24	(newborn* or new-born* or neonat* or neo-nat* or infan* or child* or adolesc* or paediatr* or pediatr* or baby* or babies* or toddler* or kid or kids or boy* or girl* or	1709151

	juvenile* or youth or teen* or pubescen* or preadolesc* or prepubesc* or preteen or tween).ti.	
25	(pediatr* or paediatr*).jw.	631543
26	or/23-25	2756871
27	22 not 26	3032
28	students/	77680
29	(young adj2 (adult or people)).tw,kf.	76425
30	((college* or undergraduate or postgraduate or university) adj2 (age* or participant* or student*)).tw,kf.	68954
31	("18 years" or "19 years" or "20 years" or "21 years" or "22 years" or "23 years" or "24 years" or "25 years" or "26 years" or "27 years" or "28 years" or "29 years" or "30 years" or "31 years" or "32 years" or "33 years" or "34 years" or "35 years").tw,kf.	500854
32	or/28-31	676462
33	22 and 32	740
34	27 or 33	3228
35	remove duplicates from 34	3220
36	("20211013" or "20211014" or "20211015" or "20211016" or "20211017" or "20211018" or "20211019" or 2021102* or 2021103* or 202111* or 202112* or 2022* or 2023*).ed,dt,ez.	2653020
37	35 and 36	817

2.5. PsycINFO (Ovid) <1806 to January Week 5 2023>

1	exp cannabis/	10533
2	"cannabis use disorder"/	673
3	marijuana usage/	3330
4	(Cannabi* or Marihuana* or Marijuana* or Hemp or Hemps or Ganja or Ganjas or Hashish or Hashishs or Bhang or Bhangs or Smoking Blunt*).ti,ab,id.	28912
5	(weed* adj3 (smok* or use* or using or abus* or dependen* or disorder*)).ti,ab,id.	19
6	or/1-5	29322
7	internet/	30782
8	exp mobile phones/	7263
9	mobile devices/	2966
10	digital gaming/	2530
11	electronic communication/	3289
12	computer mediated communication/	6605
13	exp websites/	6815
14	electronic learning/	3126
15	mobile health/	1933
16	mobile applications/	2106
17	mobile technology/	334
18	wearable devices/	657
19	computers/	10904
20	telemedicine/	7303
21	text messaging/	1415
22	Virtual reality/ or augmented reality/	11122
23	computer simulation/	4808
24	mobile learning/	833
25	online community/ or online social networks/	11160
26	social media/	14080
27	digital game-based learning/	414
28	(Web or website* or internet or echeckup or smartphone* or ((ios or android or smart or portable or cell) adj2 (device* or phone* or system* or platform* or application*)) or	349426

	mobile or online or on-line or (chat adj2 (live or based or messenger* or group* or interface or function* or forum* or transcript* or session* or log or logs or counsel* or bot or bots or support* or room or rooms)) or computer* or mhealth or m-health or ehealth or e-health or app or apps or digital or SMS or messaging or Short Message Service* or texting or ((virtual or augmented) adj2 realit*) or Serious gam* or telemedicine or telehealth* or (tele adj2 (medicine or health*)) or blog or blogs or blogging or social media* or (wearable adj2 device*) or ((electronic or digital) adj3 (communicat* or learn* or game* or gaming))).ti,ab,id.	
29	or/7-28	361663
30	6 and 29	1976
31	(newborn* or new-born* or neonat* or neo-nat* or infan* or child* or adolesc* or paediatr* or pediatr* or baby* or babies* or toddler* or kid or kids or boy* or girl* or juvenile* or teen* or youth* or pubescen* or preadolesc* or prepubesc* or preteen or tween).ti.	625008
32	(pediatr* or paediatr*).jn.	4449
33	31 or 32	626258
34	30 not 33	1635
35	exp students/ or college students/ or graduate students/ or medical students/ or postgraduate students/	297958
36	(young adj2 (adult or people)).ti,ab,id.	51110
37	((college* or undergraduate or postgraduate or university) adj2 (age* or participant* or student*)).ti,ab,id.	222841
38	("18 years" or "19 years" or "20 years" or "21 years" or "22 years" or "23 years" or "24 years" or "25 years" or "26 years" or "27 years" or "28 years" or "29 years" or "30 years" or "31 years" or "32 years" or "33 years" or "34 years" or "35 years").ti,ab,id.	88240
39	35 or 36 or 37 or 38	563709
40	30 and 39	560
41	34 or 40	1744
42	remove duplicates from 41	1741
43	("20211013" or "20211014" or "20211015" or "20211016" or "20211017" or "20211018" or "20211019" or 2021102* or 2021103* or 202111* or 202112* or 2022* or 2023*).up.	239529
44	42 and 43	283

2.6. PubMed <Oct 6 to Oct 13>

(((((Cannabi*[Title/Abstract] OR Marihuana*[Title/Abstract] OR Marijuana*[Title/Abstract] OR Hemp[Title/Abstract] OR Hemps[Title/Abstract] OR Ganja[Title/Abstract] OR Ganjas[Title/Abstract] OR Hashish[Title/Abstract] OR Hashishs[Title/Abstract] OR Bhang[Title/Abstract] OR Bhangs[Title/Abstract] OR "Smoking Blunt*"[Title/Abstract]))) AND (Web[tiab] or website*[tiab] or internet[tiab] or echeckup[tiab] or smartphone*[tiab] or ((ios[tiab] or android[tiab] or smart[tiab] or portable[tiab] or cell[tiab]) AND (device*[tiab] or phone*[tiab] or system*[tiab] or platform*[tiab] or application*[tiab])) or mobile[tiab] or online[tiab] or "on-line"[tiab] or (chat[tiab] AND (live[tiab] or based[tiab] or messenger*[tiab] or group*[tiab] or interface[tiab] or function*[tiab] or forum*[tiab] or transcript*[tiab] or session*[tiab] or log[tiab] or logs[tiab] or counsel*[tiab] or bot[tiab] or bots[tiab] or support*[tiab] or room[tiab] or rooms[tiab])) or computer*[tiab] or mhealth[tiab] or "m-health"[tiab] or ehealth[tiab] or "e-health"[tiab] or app[tiab] or apps[tiab] or digital[tiab] or SMS[tiab] or messaging[tiab] or "Short Message Service*"[tiab] or texting[tiab] or "virtual realit*"[tiab] or "augmented realit*"[tiab] or "Serious gam*"[tiab] or telemedicine[tiab] or telehealth*[tiab] or "tele medicine"[tiab] or "tele health*"[tiab] or blog[tiab] or blogs[tiab] or blogging[tiab] or "social media*"[tiab] or "wearable device*"[tiab] or ((electronic[tiab] or digital[tiab]) AND (communicat*[tiab] or learn*[tiab] or game*[tiab] or gaming[tiab]))))) AND ("2023/02/07"[Date - Create] : "3000"[Date - Create])	19

3. Results of the literature search update (February 13, 2023), for each database

Database	Results Before Duplicate Removal	Duplicates Deleted	Results to screen After Duplicate Removal	% Retained
CINAHL	349	260	89	25,5%
Cochrane Library et CENTRAL	69	53	16	23,2%
Embase	1068	675	393	36,8%
Medline	817	196	621	76,0%
PsycINFO	283	239	44	15,5%
PubMed	19	7	12	63,2%
Total	***2605***	***1430***	***1175***	***45,1%***
