# Supplementary material for: Digital Interventions for Recreational Cannabis Use Among Young Adults: Systematic Review, Meta-Analysis, and Behavior Change Technique Analysis of Randomized Controlled Studies
Source: J Med Internet Res. 2024 Apr 17;26:e55031. doi: 10.2196/55031 (PMC11063887; doi:10.2196/55031)
Supplement: Multimedia Appendix 3 [file jmir_v26i1e55031_app3.pdf]

Multimedia Appendix 3. Population, intervention, comparison, outcome, and study design strategy.

PICO ELEMENTS	CONCEPTS
**P** (Population) – Young adults	(Student*) OR (Young adult*) OR (Young people) OR (College student*) OR (Undergraduate student*) OR (Postgraduate student*) OR (University student*) OR (College age*) OR (Undergraduate age*) OR (Postgraduate age*) OR (College participant*) OR (Undergraduate participant*) OR (Postgraduate participant*) OR (University participant*) OR (18 years) OR (19 years) OR (20 years) OR (21 years) OR (22 years) OR (23 years) OR (24 years) OR (25 years) OR (26 years) OR (27 years) OR (28 years) OR (29 years) OR (30 years) OR (31 years) OR (32 years) OR (33 years) OR (34 years) OR (35 years)
**I** (Intervention) – Digital interventions on cannabis use	(Cannabi* OR Marihuana* OR Marijuana OR Hemp* OR Ganja* OR Hashish* OR Bhang* OR Smoking Blunt* OR Weed smok* OR Weed us* OR Weed abus* OR Weed dependen* OR Weed disorder*)<br>**AND**<br>(Internet OR Cellular Phone OR Text Messaging OR Smartphone OR World Wide Web OR Telehealth OR Telemedicine OR Mobile Applications OR Microcomputers OR Computer Simulation OR Augmented Reality OR Virtual Reality OR Online Services OR Online Social Networking OR Social Media OR Facebook OR Twitter OR Web OR Website* OR Internet OR Echeckup OR Smartphone OR IOS device OR IOS phone OR IOS system OR IOS platform OR IOS application OR Android device OR Android phone OR Android system OR Android platform OR Android application OR Smart device OR Smart device OR Smart phone OR Smart system OR Smart platform OR Smart application OR Portable device OR Portable device OR Portable phone OR Portable system OR Portable platform OR Portable application OR Cell device OR Cell device OR Cell phone OR Cell system OR Cell platform OR Cell application OR Mobile OR Online OR On-line OR Chat live OR Chat based OR Chat messenger* OR Chat group OR Chat interface OR Chat function* OR Chat forum* OR Chat transcript* OR Chat session* OR Chat log* OR Chat counsel* OR Chat bot* OR Chat support* OR Chat room* OR Computer* OR mHealth OR m-Health OR eHealth OR e-Health OR App* OR Digital OR SMS OR Messaging OR Short Message Service* OR Texting OR Virtual realit* OR Augmented realit* OR Serious gam* OR Telemedicine OR Telehealth* OR Tele medicine OR Tele health* OR Blog* OR Social media OR Wearable device OR Electronic communicat* OR Electronic learn* OR Electronic gam* OR Digital communicat* OR Digital learn* OR Digital gam*)
**C** (Comparison) – 2 groups or more	(Control group) OR (Wait-list) OR (Delayed-treatment) OR (Cannabis use only assessed)
**O** (Outcome) – Cannabis use	(Cannabis use frequency) OR (Cannabis use quantity) OR (Cannabis use abstinence)
**S** (Study Design) – Experimental designs	(Randomized controlled trial) OR (Cluster randomized controlled trial) OR (Pilot randomized controlled trial)
