# Supplementary material for: Digital Interventions for Recreational Cannabis Use Among Young Adults: Systematic Review, Meta-Analysis, and Behavior Change Technique Analysis of Randomized Controlled Studies
Source: J Med Internet Res. 2024 Apr 17;26:e55031. doi: 10.2196/55031 (PMC11063887; doi:10.2196/55031)
Supplement: Multimedia Appendix 4 [file jmir_v26i1e55031_app4.pdf]

Multimedia Appendix 4. Excluded studies and reasons for exclusion.

Ref ID	Reason for exclusion: Wrong type of papers (*n* = 36)
73	ACTRN. (2009). Web-based intervention for cannabis use. http://www.who.int/trialsearch/Trial2.aspx?TrialID=ACTRN12609000856213
324	Martz, M. E., Waller, R., Drislane, L. E., Bonar, E. E., Walton, M. A., Cunningham, R. M., & Blow, F. C. (2018). Therapist and computer-based brief interventions within a randomized controlled trial: effects on parallel trajectories of alcohol use, marijuana use, and anxiety. In A*lcoholism-Clinical and Experimental Research,* 42, 198A.
392	Toulet, V. D. (2015). Synthetic cannabis on the web: A mouse game! *Toxicologie Analytique et Clinique, 27*(1), 45-47.
598	NCT. (2019). Social Media Intervention for Cannabis Use in Emerging Adults. https://clinicaltrials.gov/show/NCT04187989
625	Zhang, M. W., & Ho, R. C. (2016). Smartphone for the smarter delivery of drugs, psychoeducational materials and acute intervention for at-risk users. *BMJ Innovations*, *2*(3).
628	Rooke, S., Webb, L., Albertella, L., & Copeland, J. (2015). A smartphone application for managing cannabis use. *Drug and Alcohol Dependence*, *100*(156), e192.
899	Kells, M., & Shrier, L. A. (2017). Reducing cannabis use with a real-time intervention using mobile technology. In *Handbook of Cannabis and Related Pathologies* (pp. 1101-1110). Academic Press.
954	ACTRN. (2016). A randomised controlled trial of a smartphone application for people wanting to reduce or quit their use of cannabis. https://www.who.int/trialsearch/Trial2.aspx?TrialID=ACTRN12616000622404
1099	NCT. (2019). Project RAD: a Brief Intervention for Young Adult Alcohol or Marijuana Users. https://clinicaltrials.gov/show/NCT03801096
1243	Bedrouni, W. (2018). On the use of digital technologies to reduce the public health impacts of cannabis legalization in Canada. *Canadian Journal of Public Health*, *109*, 748-751.
1611	NCT. (2017). Personalized Health Assessment Related to Medications (Project PHARM). https://clinicaltrials.gov/show/NCT03037476
1612	NCT. (2019). Personalized Feedback Intervention to Reduce Risky Cannabis Use. https://clinicaltrials.gov/show/NCT04060602
1706	Palfai, T., Saitz, R., & Winter, M. R. (2015). Moderators of electronic screening and brief intervention for marijuana among students in a health center: eCHECKUP TO GO bolsters efforts of those who are already trying to change. *Drug and Alcohol Dependence*, *100*(156), e170.
1822	Putnam, J., Skinner, K., & Kiger, A. (2018). Marijuana and Missouri college health promotion: an exploratory study. *Missouri Nurse*, 12-15.
1967	ACTRN. (2018). Keep it Real: a web-based program for psychotic-like experiences and cannabis use. http://www.who.int/trialsearch/Trial2.aspx?TrialID=ACTRN1261800110721
2045	(2015). Interactive App Teaches Skills for Conversations about Alcohol and Marijuana Use. *Colorado Nurse, 118*, 5.
2129	ACTRN. (2018). Implementation and effectiveness of an early intervention program (QuikFix) for young people experiencing alcohol and other drug-related harm. http://www.who.int/trialsearch/Trial2.aspx?TrialID=ACTRN12618001010280.
2250	WHO. ICAN: an online training to reduce cannabis use. http://www.who.int/trialsearch/Trial2.aspx?TrialID=NL7668
2421	NCT. (2015). Internet-Based Treatment for Individuals With Regular Cannabis Use: a Randomized Controlled Trial. https://clinicaltrials.gov/show/NCT02408640
2422	Drks. (2014). Internet based Social Norms Intervention for substance use prevention for Students'-study. http://www.who.int/trialsearch/Trial2.aspx?TrialID=DRKS00007635.
2445	Smelror, R. E., Bless, J. J., Hugdahl, K., & Agartz, I. (2019). Feasibility and acceptability of using a mobile phone app for characterizing auditory verbal hallucinations in adolescents with early-onset psychosis: Exploratory study. *JMIR Formative Research*, *3*(2), e13882.

2556	ISRCTN. (2010). Evaluation of an internet-based one-time counselling intervention for young cannabis- or alcohol-users. http://www.who.int/trialsearch/Trial2.aspx?TrialID=ISRCTN79857771.
2557	Elliott, J. C. (2012). *Evaluation of a Web-based intervention for college marijuana use* (Doctoral dissertation, Syracuse University).
2724	Collier, R. (2017). Health Canada to warn young people of cannabis risks in campaign. Canadian Medical Association Journal, 189(41), E1297. https://doi.org/10.1503/cmaj.109-5506
2766	Blow, F., Bohnert, A. S., Ignacio, R., Barry, K., Ilgen, M. A., Booth, B. M., ... & Walton, M. (2015). Efficacy of computer and therapist brief interventions for drug users. *Drug and Alcohol Dependence*, *100*(156), e21.
2788	Amann, M., Haug, S., Wenger, A., Baumgartner, C., Ebert, D. D., Berger, T., ... & Schaub, M. P. (2018). The effects of social presence on adherence-focused guidance in problematic cannabis users: protocol for the CANreduce 2.0 randomized controlled trial. *JMIR Research Protocols*, *7*(1), e9484.
2957	NCT. (2013). An Exercise App to Reduce Young Adults' MJ Use. https://clinicaltrials.gov/show/NCT01863095
3068	Gagnon, H., Côté, J., Tessier, S. & April, N. (2012). Development of a Web platform to reduce cannabis use among youth who attend adult education centres. *Drogues, Santé et Société, 11*(2), 1-17.
3427	(2017). CDPH Offering Online Cannabis Guidance. *Occupational Health & Safety News,* 1-2.
3456	Schneeberger, D., Bonar, E. E., Bauermeister, J., Young, S., Blow, F. C., Bourque, C., ... & Walton, M. A. (2019). Developing a motivational interviewing fidelity coding scheme for alcohol interventions delivered by e-health coaches on social media. A*lcoholism-Clinical and Experimental Research,* 43, 79A.
3472	ISRCTN. (2016). CANreduce 2.0 - comparing two differently optimized versions of a web-based self-help program to reduce cannabis use with each other and a waiting list. http://www.who.int/trialsearch/Trial2.aspx?TrialID=ISRCTN11086185.
3612	Schaub, M. P., Haug, S., Wenger, A., Berg, O., Sullivan, R., Beck, T., & Stark, L. (2013). Can reduce-the effects of chat-counseling and web-based self-help, web-based self-help alone and a waiting list control program on cannabis use in problematic cannabis users: a randomized controlled trial. *BMC Psychiatry*, *13*, 1-10.
3637	Jones, H. (2013). Brief Interventions for Cannabis Use: Increasing Engagement and Motivation for Change in Young People. *Turkish Archives of Pediatrics, 48*(Suppl 2), 125.
3745	Copeland, J., Rooke, S., & Gibson, L. (2017). Comparison of brief versus extended feedback in an online intervention for cannabis users: A randomised controlled trial. *Drug and Alcohol Dependence*, *100*(171), e45.
4179	Gardner, L. (2018). Internet-Based Prevention for Alcohol and Other Drugs: An Overview of the Universal Climate Schools Prevention Programs. In *65th Annual Meeting*. AACAP.
4501	Llanes, K. D. (2020). *Comparing the Language of Computer-mediated Versus Face-to-face Motivational-type Interviews* (Doctoral dissertation, The University of Texas at El Paso).
