# Supplementary material for: Digital Interventions for Recreational Cannabis Use Among Young Adults: Systematic Review, Meta-Analysis, and Behavior Change Technique Analysis of Randomized Controlled Studies
Source: J Med Internet Res. 2024 Apr 17;26:e55031. doi: 10.2196/55031 (PMC11063887; doi:10.2196/55031)
Supplement: Multimedia Appendix 5 [file jmir_v26i1e55031_app5.pdf]

Multimedia Appendix 5. Study and participant characteristics.

Study	Country	Study aim	Study design	Intervention condition	Comparator(s)	Participant characteristics			
						Population	Baseline sample (*n*)	Mean age (*SD*)	Gender (female)
Baumgartner et al., 2021	Switzerland	"To investigate whether intervention effectiveness and program adherence can be increased by implementing adherence-focused guidance and emphasizing the social presence factor of a personal eCoach when compared with a general support team implementation"	RCT	**Brief description:** Minimally guided internet-based self-help intervention with a personal online coach (eCoach) **Name:** CANreduce 2.0 with social presence	**Active comparator:** CANreduce 2.0 with an impersonal service team **Passive comparator:** Waiting list (access to internet as usual)	Adult cannabis users; 18+ years old; who reported CU at least once weekly over the last 30 days	575	28.3 (7.9)	29.4%
Bonar et al., 2022	USA	"To develop and pilot-test a new 8-week social media intervention for emerging adults' CU"	Pilot RCT	**Brief description:** Social media-delivered, MI and CBT-based intervention targeting CU among emerging adults **Name:** NR[a]	**Passive comparator:** Attention control (Facebook private group in which staff posted manual-based content and brief responses to participant comments, unrelated to CU or mental health and not using therapeutic techniques)	Emerging adults (ages 18–25); who used cannabis ≥ 3 times/week in the past-month	149	21.0 (2.2)	55.7%
Bonar et al., 2023	USA	"To develop and evaluate a physical activity-focused social-media delivered cannabis intervention for emerging adults"	Pilot RCT	**Brief description:** Social media-delivered intervention for emerging adults who use cannabis that focuses on physical activity, and includes MI and CBT cannabis intervention content (physical activity + cannabis) **Name:** NR[a]	**Active comparator:** Facebook group with e-coaches posting content aligning with physical activity-focused topics (physical activity only) **Passive comparator:** Attention control (manualized content posted in the Facebook group unrelated to physical	Emerging adults (ages 18–25); who reported past-month cannabis consumption ≥ 3 times; ability to safely engage in exercice	60	21.7 (2.3)	63.3%

					activity, CU, mental health)				
Buckner et al., 2020	USA	"To pilot test a newly developed PFI for negative affect and cannabis (PFI-NAC) designed to reduce cannabis use, teach quit strategies and other skills to manage cravings, and teach skills to manage negative affect"	Pilot RCT	**Brief description:** Online PFI for negative affect and cannabis (PFI-NAC) **Name:** NR[a]	**Passive comparator:** Assessment only[b]	Adult cannabis users; 18+ years old; who reported past month CU	63	CG = 19.3 (1.0) IG = 18.9 (1.8)	84.1%
Copeland et al., 2017	Australia	"To test the short-term effectiveness of brief and extended personalized feedback as part of the online intervention program, *Grassessment*, in reducing CU and dependence severity"	RCT	**Brief description:** Brief, online self-complete motivational enhancement intervention for cannabis users that provides individualized extended feedback regarding use, motives, and harms **Name:** *Grassessment: Evaluate Your Use of Cannabis* + extended feedback	**Active comparator:** Screening + brief PNF (brief feedback version of the *Grassessment* program)	Adult cannabis users from the general community; 18+ years old; who expressed desire to reduce or quit their cannabis	287	Median age (range): 26 (18-65)	38.0%
Côté et al., 2018	Canada	"To evaluate the efficacy of the Web-based tailored intervention to reduce cannabis use (primary outcome) by bolstering intention (secondary outcome) to abstain from use"	RCT	**Brief description:** Web-based tailored intervention to reduce CU **Name:** *Ma réussite, mon choix*	**Passive comparator:** Assessment only[b]	Young people attending adult education centers; 18-24 years old	588	19.8 (1.9)	54.3%
Cunningham et al., 2021	Canada	"To examine the efficacy of a brief, online personalized normative feedback intervention in a large, non-college sample of adults in Canada following national legalization of recreational CU"	RCT	**Brief description:** Brief, online personalized normative feedback intervention for risky CU to motivate change (personalized feedback report + educational materials about risky CU) **Name:** NR[a]	**Active comparator:** Educational materials about risky CU only	Non-college adults; 18+ years old; with a moderate risk associated with CU (score of 4 or more on the ASSIST)	744	CG = 36.3 (12.6) IG = 35.3 (13.0) [1]	56.3%

[1] Mean age for all participants not reported. This study was included in the review based on the mean age of intervention group participants.

Elliott et al., 2012	USA	"To evaluate the efficacy of the Marijuana eCHECKUP TO GO in correcting descriptive norms, correcting injunctive norms, and preventing initiation of marijuana use in a group of college-age abstainers"	RCT	**Brief description:** Brief, norm-correcting, web-based prevention, and intervention program for individuals currently abstaining from marijuana **Name:** The Marijuana eCHECKUP TO GO (e-TOKE) for Universities and Colleges	**Passive comparator:** Assessment only[b]	University students; 18+ years old; who did not report past month CU (abstainers)	245	20.5 (2.7)	73.0%
Elliott et al., 2014	USA	"To evaluate the short-term effectiveness of eCHECKUP TO GO in changing marijuana involvement and perceived norms in undergraduates"	RCT	**Brief description:** Brief, self-paced, web-based marijuana educational program that incorporates personalized feedback and norm correction **Name:** The Marijuana eCHECKUP TO GO (e-TOKE) for Universities and Colleges	**Passive comparator:** Assessment only[b]	College students; who reported past month CU	317	NR	52.0%
Goodness et al., 2020	USA	"To assess the feasibility of implementing and testing the efficacy of a web-based screening and brief intervention to reduce marijuana use and consequences among graduate student presenting to a student health center"	RCT	**Brief description:** Electronic screening and brief intervention to reduce marijuana use and consequences with a booster session (additional feedback on CU at the 3-month timepoint following assessment) **Name:** eCHECKUPTOGO-cannabis + booster intervention	**Active comparator:** Baseline assessment + minimal general health information[c]	Graduate/ professional students; who reported using cannabis at least monthly over the past 90 days who presented themselves to a student health center	49	25.53 (2.42)	51.0%
Jonas et al., 2018	Germany	"To examine whether the effectiveness of Quit the Shit (QTS) is reduced by shortening the program or by removing the chat-based counseling option"	RCT	**Brief description:** Therapist-guided internet intervention for cannabis users with chat-based	**Active comparators:** ***Group 1:*** QTS version 2 (chat and 28 days)	Adult cannabis user; 18+ years old	534	27.5 (7.0)	34.3%

				(synchronous) and time-lagged (asynchronous) counseling **Name:** Quit the Shit (original version, chat, and 50 days long)	***Group 2:*** QTS version 3 (no chat and 50 days) ***Group 3:*** QTS version 4 (no chat and 28 days)				
Lee et al., 2010	USA	"To evaluate preliminary efficacy of a brief, web-based personalized feedback intervention for marijuana-using students transitioning to college and to examine contemplation for change and family history of drug problems as potential moderators of intervention efficacy"	RCT	**Brief description:** Brief, web-based PFI for at-risk marijuana users transitioning to college **Name:** NR[a]	**Passive comparator:** Assessment only[b]	University students; 17-19 years old; who reported past 3-months CU	341	18.03 (0.31)	54.6%
Palfai et al., 2014	USA	"To test the feasibility of procedures to screen students for marijuana use in student health services and test the efficacy of a web-based intervention designed to reduce marijuana use and consequences"	Pilot RCT	**Brief description:** Web-based screening and brief intervention for marijuana users **Name:** The Marijuana eCHECKUP TO GO (e-TOKE)	**Active comparator:** Baseline assessment + feedback on general health-related behaviors[d]	Undergraduate students presenting to a university health center; who reported using marijuana at least monthly over the past 90 days	123	Reported by site and intervention condition: On-site: CG = 20.33 (1.27) IG = 19.33 (1.14) Off-site: CG = 19.62 (1.20) IG = 19.35 (1.20)	Reported by site and interventio n condition: On-site: CG = 58.0% IG = 56.0% Off-site: CG = 54.0% IG = 62.0%
Riggs et al., 2018	USA	"To pilot-test the efficacy of an adapted personalized feedback + protective behavioural strategies version of Marijuana eCHECKUP TO GO in reducing heavy marijuana use among college students"	RCT	**Brief description:** Web-based marijuana use intervention providing university-specific personalized feedback with normative information and protective behavioural strategies **Name:** Adapted version of the Marijuana	**Active comparator:** Healthy stress management[e]	University students; 18+ years old; recreational marijuana users (i.e., nonmedical use); who reported typical marijuana use of at least twice per week	298	19.97 (2.0)	49.0%

				eCHECKUP TO GO (e-TOKE)					
Rooke et al., 2013	Australia	"To test the effectiveness of the program in assisting individuals who wished to reduce or stop their cannabis use"	RCT	**Brief description:** Fully self-guided web-based treatment program for CU and related problems **Name:** Reduce Your Use: How to Break the Cannabis Habit	**Active comparator:** 6 modules of web-based educational information on cannabis	Adult cannabis users; 18+ years old; who reported past month CU and who expressed a desire to stop or reduce CU	225	CG = 30.18 (9.62) IG = 31.88 (9.85)	38.7%
Schaub et al., 2015	Switzerland	"To investigate and compare the efficacy of web-based self-help interventions—in combination with or without tailored chat counseling based on CBT, MI, and behavioural self-management—in reducing cannabis use in problematic cannabis users"	RCT	**Brief description:** Web-based self-help intervention with tailored chat counseling to reduce CU **Name:** CANreduce 1.0	**Active comparator:** CANreduce 1.0 without tailored chat counseling **Passive comparator:** Waiting list	Adult cannabis users; 18+ years old; who reported using cannabis at least once a week over the 30 days prior to study entry	308	29.8 (10.0)	24.7%
Sinadinovic et al., 2020	Sweden	"To investigate the effects of a web-based treatment program with therapist guidance for adults and adolescents with regular cannabis use from the general population"	RCT	**Brief description:** Web-based treatment program with therapist guidance designed to help regular cannabis users to reduce or end their CU **Name:** *Cannabishjälpen*	**Passive comparator:** Waiting list	Adult cannabis users from the general population; 16+ years old; who had used cannabis at least once a week during the last 6 months	303	27.4 (7.2)	32.7%
Tossman et al., 2011	Germany	"To assess the effectiveness of QTS, with the use frequency and quantity being primary outcomes"	RCT	**Brief description:** Web-based counselling program developed to help young people to quit or reduce their CU significantly **Name:** Quit the Shit	**Passive comparator:** Waiting list	Adult cannabis users; who expressed the wish to reduce or to cease CU	1 292	24.7 (6.8)	29.5%
Walukevich-Dienst et al., 2019	USA	"To test intervention effects (moderated by gender) of an online, university-specific PFI for high-risk cannabis users (i.e., past-month cannabis users with at	RCT	**Brief description:** Online PFI for cannabis-using college students with	**Active comparator:** PNF only	Undergraduate students; who endorsed past-month CU and who experienced at least	204	19.83 (1.43)	77.0%

least one recent cannabis-related problem)”	additional information on CU risks<br>**Name:** NR[a]	one CU-related problem in the past 3 months

ASSIST = Involvement Screening Test; CBT = cognitive behavioural therapy; CG = control group; CU = cannabis use; IG = intervention group; MI = motivational interviewing; NR = not reported; PFI = personalized feedback intervention; PFI-NAC = personalized feedback intervention for negative affect and cannabis; PNF = personalized normative feedback; QTS = ‘Quit the Shit’; RCT = randomized controlled trial; SD = standard deviation.

[a] No specific intervention name reported.

[b] Participants were asked to complete baseline and follow-up assessments (or baseline only) and were not given any feedback.

[c] Participants completed a series of questions about CU and other health-related behaviors. Then, they received minimal electronic feedback on recommended hours of sleep, exercise, and diet, based on their baseline responses. No feedback on CU was provided.

[d] Participants were given minimal general health feedback regarding recommended guidelines for sleep, exercise, and nutrition.

[e] Participants were provided with strategies for healthy stress management (e.g., deep breathing, mindfulness, exercise).
