# Supplementary material for: Digital Interventions for Recreational Cannabis Use Among Young Adults: Systematic Review, Meta-Analysis, and Behavior Change Technique Analysis of Randomized Controlled Studies
Source: J Med Internet Res. 2024 Apr 17;26:e55031. doi: 10.2196/55031 (PMC11063887; doi:10.2196/55031)
Supplement: Multimedia Appendix 6 [file jmir_v26i1e55031_app6.pdf]

Multimedia Appendix 6. Description of intervention characteristics in the included articles.

Study	Brief description	Purpose of intervention	Technology	Theory[a]	Treatment approaches[b]	Duration and/or frequency	Salient features		
							Tailoring	Prompts and reminders	Human involvement
**Study of *Cannabishjälpen***									
Sinadinovic et al., 2020	Web-based treatment program with therapist guidance for regular cannabis users	Help reduce or end CU	**Web-based** Online modules with automated feedback computer-mediated communication	NR	- Psychoeducational information developed from a manual-based treatment program for chronic cannabis users entitled 'A way out of fog' (Lundqvist, 1997)<br>- Training and exercises grounded on principles of CBT and MI	**Frequency:** All modules (*n* = 13) were available to the participants at once, with the recommendation of doing one or two modules per wk<br>**Duration:** 6 wks	**Yes**<br>Personal feedback based on the participant's consumption calendar entries	**Yes**<br>Automated recommendations about which modules to work with	**Yes**<br>Participants had the opportunity to communicate with the therapist throughout the entire program, on their own terms
**Studies of *CANreduce***									
Baumgartner et al. 2021	Minimally guided internet-based self-help intervention with social presence (CANreduce 2.0)	Reduce CU	**Web-based** Online modules with email feedback and computer-mediated communication with an online coach (eCoach)	Adherence-focused guidance enhancement based on the supportive-accountability model of guidance (Mohr et al., 2011)	- Behavioral activation approach (Kanter et al., 2012)<br>- CBT (Jaffee & D'Zurilla, 2009)<br>- MI (Miller & Rollnick, 1991)<br>- Relapse prevention (Marlatt & Donovan, 2005)<br>- Social problem-solving approach (Platt & Husband, 1993)	**Frequency:** NR<br>**Duration:** 6 wks to complete 8 modules	**Yes**<br>Email feedback based on the participant's CU diary and potential questions raised by the modules	**Yes**<br>Weekly semiautomated motivational and adherence-focused guidance-based email feedback<br>Emails also included module suggestions	**Yes**<br>**Group with social presence:** semiautomated eCoach (fictional companions who appeared at key points in the modules via short videos to encourage reflection)<br>**Group with an impersonal service team:** anonymous semiautomated support team, with no pictures, videos, or any other kind of social presence

Schaub et al., 2015	Web-based self-help intervention with tailored chat counseling (CANreduce 1.0)	Reduce CU in problematic users	**Web-based** Online modules with automated motivational emails and tailored online chat counseling within the website	NR	- Classical CBT for treating cannabis dependance (Steinberg et al., 2005)<br>- MI (Miller & Rollnick, 2002)	**Frequency:** First chat session between wk 1 and 2, and second chat session between wk 4 and 6 **Duration:** Access to the intervention website (8 modules) was offered for 6 wks; the scheduled counseling chat sessions lasted 20 to 30 min each	**Yes** Structure of the chat session was made more flexible and more dependent on the participants' needs and considered the data the participants entered the self-help intervention (i.e., consumption diary)	**Yes** Weekly automated motivational emails to remind the user to log in	**Yes** Up to two individual chat sessions with counselors that received quarterly supervision sessions and consisted of trained MI counselors, mainly psychologists or psychiatrists with advanced or completed further education, with at least one year of experience in treating cannabis-abusing patients face to face
**Study of *Grassessment: Evaluate Your Use of Cannabis***									
Copeland et al., 2017	Brief, online, self-complete motivational enhancement intervention for cannabis users by assessing and presenting personalized and normative information	Reduce participants' CU and dependance severity	**Web-based** Website of the Grassessment program that includes personalized feedback, both in real time as participants move through the assessment via infographics, and via a summary screen and email at the completion of the program	NR	- Brief motivational enhancement approach<br>- Psychoeducational information	**Frequency:** NR **Duration:** NR	**Yes** Personalized extended feedback regarding use, motives, and harms, and Individualized referral support (links to specialist services at program completion based on participants ideal treatment option)	**No**	**No**
**Studies of *The Marijuana eCHECKUP TO GO (e-TOKE)***									

Elliott et al., 2012	Brief, norm-correcting, web-based prevention, and intervention program for individuals currently abstaining from marijuana	Help college students think carefully about the decision of whether to use marijuana	**Web-based** Any computer with Internet access	NR	NR	**Frequency:** The program allows for participants to complete the program at any pace **Duration:** ≈20 min	**Yes** Personalized feedback on marijuana use, annual money spent on substances, suggestions for campus resources that fits the participant's needs, and possible steps for decreasing use	**No**	**No**
Elliott et al., 2014	Brief, self-paced, web-based marijuana educational program that incorporates personalized feedback and norm correction	Prompt self-reflection and consideration of decreased use	**Web-based**[c]	NR	NR	**Frequency:** NR **Duration:** Participation typically takes 20 min; thorough review of all material may take 45 min	**Yes** Participants received feedback based on their marijuana use (e.g., norms and annual expense of substance use), personalized campus resource information, and tips to decrease use	**No**	**No**
Goodness et al., 2020	Electronic screening and brief intervention with additional feedback on CU at the 3-month timepoint following assessment (eCHECKUPTOGO-cannabis + booster intervention)	Reduce marijuana use and consequences	**Web-based** Commercially available electronic intervention	NR	Participants were provided with harm- and frequency-reduction strategies	**Frequency:** Participants received the intervention on two occasions, at baseline and following the 3-month follow-up outcome assessment (as a "booster" session) **Duration:** NR	**Yes** Personalized feedback about CU, including costs, descriptive norms, risks, consequences, and potential alternative activities	**Yes** At the 3-month "booster" session, participants were encouraged to compare their current CU and consequences to their previous patterns of use, as well as to consider progress on the pursuit of important life goals	**No**

Palfai et al., 2014	Web-based screening and brief intervention for marijuana users	Reduce marijuana use and consequences	**Web-based** Commercially available electronic intervention	NR	NR	**Frequency:** NR **Duration:** NR	**Yes** Participants received detailed personalized feedback about their marijuana use including costs, norms, risks, consequences, and alternative activities	**No**	**No**
Riggs et al., 2018	Marijuana Web-based marijuana use intervention providing university-specific personalized feedback with normative information and protective behavioural strategies (Adapted version of the Marijuana eCHECKUP TO GO)	Reduce the harms associated with heavy marijuana use by correcting normative perceptions of marijuana use	**Web-based**[c]	NR	- Protective behavioral strategies-approaches to marijuana (Prince et al., 2013)	**Frequency:** NR **Duration:** NR	**Yes** Participants received personalized feedback regarding personal marijuana use and perceptions of marijuana use norms versus actual use prevalence at their university and nationally	**Yes** Participants were encouraged to use protective behavioral strategies with a list of potential change strategies related to marijuana use	**No**

Study of *Ma réussite, mon choix*

Côté et al., 2018	Web-based tailored intervention	Reduce CU by strengthening the intention to abstain	**Web-based** Web platform using a computer algorithm to establish the participants' user profiles	Theory of Planned Behavior (Ajzen, 1985) The intervention focused on three determinants: attitude, perception of control, and habit (i.e., frequency of use)	- PFI<br>- Psychoeducational messages targeting the intervention determinants to bolster a positive attitude and a sense of control regarding cannabis abstinence	**Frequency:** 5 brief visits to the Web platform (≈10 min each) at 2-wk intervals for 6 wks **Duration:** ≈50 min	**Yes** Seven user profiles were generated based on frequency of CU in the past month/year and intention to abstain in the coming month; each profile corresponding to a tailored intervention sequence Personalized reinforcement message (i.e., positive feedback on behavior) provided at each visit (up to 16 messages)	**Yes** A youth worker at each center reminded participants when to log into the intervention Web platform	**Yes** At each visit, an educational message was delivered by a credible character in short video clips. This character resembled a young adult that might have used cannabis and decided to quit
**Studies of *Quit the Shit (QTS)***									
Jonas et al., 2018	Therapist-guided internet intervention for cannabis users with chat-based (synchronous) and time-lagged (asynchronous) counseling	Reducing problematic CU	**Web-based** Free and anonymous program website with personalized online chat counseling	QTS in general is based on principles of self-regulation and self-control (Miller & Heather, 1986)	The weekly feedbacks are based on the solution-focused approach (De Shazer et al., 2007) and MI (Miller & Rollnick, 1991)	**Frequency:** Counselor feedback is timed weekly **Duration:** Comprises of 3 consecutive phases: 1) admission mandatory chat (prescheduled appointment in a one-to-one live chat with a counselor for ≈50 min); 2) login area activated for 50 days; and 3) concluding chat (duration NR)	**Yes** Participants received up to seven personalized feedback messages once a wk by their counselor on their entries in diary and coping exercises	**No**	**Yes** Real-time-counseling via text-chat and personalized feedback with the same counselor throughout the whole program to discuss the current CU, the psychosocial situation, and the counseling process as such

Tossman et al., 2011	Web-based counselling program for cannabis users	Help young people to quit or reduce their CU significantly	**Web-based** Program website with personalized online chat counseling using a secure connection to the server via https-protocol Integrated into the drug prevention website www.drugcom.de	Program structure and counseling are based on the principles of self-regulation and self-control (Miller & Heather, 1986)	A solution-focused approach (De Shazer et al., 2007) is applied in the intervention	**Frequency:** NR **Duration:** Comprises 4 consecutive phases: 1) registration (online questionnaire); 2) admission one-on-one chat carried out by a counselor (≈50 min); 3) online diary of QTS activated for 50 days; and 4) concluding chat (duration NR)	**Yes** Weekly feedback provided by counselors about consumption, psychosocial situation of the participant, and to the counseling process as such	**No**	**Yes** Real-time-counseling via text-chat and personalized feedback with the same counselor throughout the whole program (one of four qualified psychotherapists)
**Study of *Reduce Your Use: How to Break the Cannabis Habit***									
Rooke et al., 2013	Fully self-guided web-based treatment program for CU and related problems	Assisting individuals who wished to reduce or stop their CU	**Web-based** Online modules using a website with password-protected access	Concept of Behavioral Self-Management (Copeland et al., 2001)	- Based on a face-to-face brief treatment previously found to be effective for problematic cannabis use (Copeland et al., 2001), which was informed by the principles of CBT and MI<br>- Web-adaptation was informed by other web-based interventions targeting substance use that used automated feedback (Linke et al., 2008)	**Frequency:** The website contains 6 core modules, which are undertaken sequentially at intervals chosen by the participant (1 module recommended per wk) **Duration:** ≈6 wks	**Yes** Personalized feedback on the participant's progress (changes in CU and related factors, goal setting, weekly expenditure on cannabis) The website also features a personalized folder for the participant	**Yes** Weekly automatically generated encouragement emails	**No**
**Studies of social media-delivered cannabis interventions[d]**									

Bonar et al., 2022	Social media-based, MI and CBT-based intervention targeting CU among emerging adults	To help emerging adults who frequently use cannabis increase well-being and reduce risky behaviors	**Social media-delivered** Constantly accessible social media-based platform (Facebook private group)	NR	- MI and CBT-based approaches<br>- Harm reduction approach<br>- Psychoeducation about CU potency and risks	**Frequency:** 56 days (8 wks)<br>**Duration:** Daily, electronic health coaches (e-coaches) posted content on the Facebook private group page (≈6 posts/day)	**No**	**No**	**Yes**<br>Master's-level e-coaches with relevant backgrounds (e.g., social work) posting online cannabis-related content and responding to participants' comments<br>E-coaches completed online MI training modules, attended a two-day MI training, and received study-specific trainings about cannabis, cognitive-behavioral skills for cannabis reduction, social media intervention delivery, and risk management

Bonar et al., 2023	Social media-delivered intervention for emerging adults who use cannabis that focuses on physical activity and includes MI and CBT cannabis intervention content (physical activity + cannabis)	Reducing CU	**Social media-delivered** Constantly accessible social media-based platform (Facebook private group)	NR	- MI and CBT-based approaches for CU<br>- Harm reduction approach<br>- Psychoeducation about CU (e.g., cannabis consequences, cannabis impact on relationships, managing triggers)<br>- Weekly themes incorporating content and topics from prior lifestyle interventions (Wang et al., 2017), including topics aligned with physical activity-focused elements of the gold-standard Diabetes Prevention Program (Sepah et al., 2014)	**Frequency:** 56 days (8 wks)<br>**Duration:** Daily, electronic health coaches (e-coaches) posted content on the Facebook private group page (≈6 posts/day)	**Yes**<br>Before each intervention post, e-coaches confirmed relevance of content, making edits to reflect current topics (e.g., incorporating something a participant shared earlier)	**No**	**Yes**<br>Three bachelor's and master's-level e-coaches with relevant experience and training who posted online physical activity and cannabis-related content, and responded to participants' comments<br>E-coaches completed online MI training modules, study-related training in delivering MI on social media, study-specific trainings about cannabis and physical activity, cognitive-behavioral skills for changing cannabis and health behaviors, and risk management
**Studies of PFIs**[d]									
Buckner et al., 2019	Online PFI for negative affect and cannabis (PFI-NAC)	Reduce CU, teach quit strategies and other skills to manage cravings, and teach skills to manage negative affect	**Web-based** The entire intervention was delivered via Qualtrics	NR	- PFI<br>- Psychoeducation on general information about cannabis, several quit strategies, coping with craving strategies, negative affect, increasing social support, and positive journaling	**Frequency:** NR<br>**Duration:** NR	**Yes**<br>Personalized feedback using data from participants' responses to assessments completed at the baseline concerning aspects of cannabis (e.g., carbon monoxide levels, financial costs of cannabis) and their CU and negative affect	**No**	**No**

Cunningham et al., 2021	Brief, online personalized normative feedback intervention for risky CU (personalized feedback report + educational materials about risky CU)	Motivate change towards risky CU	**Internet-based** Emails sent to participants to access the study website (link to complete baseline assessment used to provide personalized feedback), and offer a personalized feedback final report accompanied with educational material	NR	- Updated version of an existing personalized normative feedback intervention for risky cannabis (Cunningham & van Mierlo, 2009)<br>- Feedback on CU consequences based on a feedback framework developed by Bertholet et al. (2015) in their PFI for alcohol<br>- Psychoeducational material based on the “Canada's Lower-Risk Cannabis Use Guidelines”	**Frequency:** NR<br>**Duration:** NR	**Yes** Participants were provided with a personalized feedback final report allowing them to compare their own CU with others of the same gender and age group, and based on their personal score on the Marijuana Problems Scale and the ASSIST cannabis subscales	**No**	**No**
Lee et al., 2010	Brief, web-based PFI for at-risk marijuana users transitioning to college	Reduce marijuana use and prevent from marijuana-related consequences in college students	**Web-based** The feedback was primarily text based but incorporated pictures to enhance interest and appeal as well as figures/graphs representing normative information and comparisons	NR	- MI approach described by Miller and Rollnick (1991)<br>- Brief PFI approach for alcohol prevention (Brief Alcohol Screening and Intervention for College Students) pioneered by Marlatt et al. (1998)<br>- Also informed by recent work regarding PFI for adolescent marijuana smokers (Walker et al., 2006) and studies of computerized normative feedback for alcohol prevention<br>- Skills training tips for avoiding marijuana and making changes in one’s use	**Frequency:** On completion of the baseline survey, PFI participants could immediately view feedback online<br>**Duration:** Participants could return to view feedback on the web for 3 months	**Yes** Individual personalized feedback based on baseline information (i.e., marijuana use, perceived and actual descriptive norms for marijuana use, self-reported negative consequences, and perceived pros and cons of using marijuana)	**No**	**No**

Walukevich-Dienst et al., 2019	Online PFI for cannabis-using college students with additional information on CU risks	Reduce cannabis-related problems	**Web-based**[c]	NR	- Cannabis PFI for undergraduates containing PNF on CU norms, modified from alcohol PFIs (Miller et al., 2013)<br>- Problem-focused approach<br>- Psychoeducation (information about risks and consequences associated with CU)	**Frequency:** NR<br>**Duration:** NR	**Yes**<br>PNF concerning participants' past month CU and cannabis problems, and personalized risks for CU disorder	**No**	**No**

BCT = cognitive behavioral therapy; CU = cannabis use; eCoach = personal online coach; MI = motivational interviewing; min = minutes; NR = not reported; PFI = personalized feedback intervention; PFI-NAC = personalized feedback intervention for negative affect and cannabis; PNF = personalized normative feedback; QTS = Quit the Shit; wk = week.

[a] Theories are used to inform intervention design and components, and allow to highlight the determinants (e.g., attitude, perception of control, habits, intention to change) to be targeted.

[b] Treatment approaches refer to strategies or methods (e.g., BCT, MI, personalized feedback) used within a given intervention and derive from theories.

[c] No detailed information reported about the technology used.

[d] No specific intervention name reported.
