# Supplementary material for: Digital Interventions for Recreational Cannabis Use Among Young Adults: Systematic Review, Meta-Analysis, and Behavior Change Technique Analysis of Randomized Controlled Studies
Source: J Med Internet Res. 2024 Apr 17;26:e55031. doi: 10.2196/55031 (PMC11063887; doi:10.2196/55031)
Supplement: Multimedia Appendix 7 [file jmir_v26i1e55031_app7.pdf]

Multimedia Appendix 7. Summary of methodological characteristics and major findings of the included studies categorized by intervention name

Study	Methodological characteristics					Attrition rates (%)	Major findings
	Study groups	Outcome variable[a]	Outcome measures	Data collection time points	Analytical approach		
**Study of *Cannabishjälpen***							
Sinadinovic et al., 2020	**IG:** Web-based treatment program with therapist guidance designed to help regular cannabis users to reduce or end their CU<br>**CG:** Waiting list	CU	- Self reported measure of CU frequency, by number of days without CU in the past 7 days using TLFB method (Sobell & Sobell, 1992)<br>- Self-reported measure of CU quantity, by estimated grams of cannabis consumed in the past 7 days	Baseline & 3 mo after recruitment to the study	Time by group interaction effects were modeled using GEE, which include all available data (consistent with the ITT principle) and use the appropriate family function (i.e., Poisson, Gaussian or binomial)	59.1% (*n* = 179/303)	- GEE models (ITT analyses) revealed no time by group effects on number of days without CU in the past 7 days (Poisson; *B* = 0.09; *SE* = 0.19; *p* = 0.63) or on estimated grams of cannabis consumed in the past 7 days (Poisson; *B* = -0.27; *SE* = 0.18; *p* = 0.12)<br>- Secondary analysis (i.e., excluding participants who sought other treatment) revealed that the IG showed significantly greater reductions over time in gram cannabis used past wk (Poisson; *B* = -0.42; *SE* = 0.19; *p* = 0.02)
		Cannabis-related consequences	Self-reported measure of CU problems using the assessment instrument Cannabis Abuse Screening Test (CAST) (Legleye, 2018; Legleye et al., 2007)				- GEE models revealed no time by group effects on CAST score (Gaussian; *B* = -1.63; *SE* = 0.9; *p* = 0.07)<br>- Secondary analysis (i.e., excluding participants who sought other treatment) revealed that the IG showed significantly greater reductions over time on CAST score (Gaussian; *B* = -1.63; *SE* = 0.9; *p* = 0.07)
**Studies of *CANreduce* (*n* = 2)**							
Baumgartner et al., 2021	**IGs:**<br>***Group 1:*** CANreduce 2.0 with social presence (minimally guided internet-based self-help intervention with a personal online coach [eCoach])<br>***Group 2:*** CANreduce 2.0 with a general support	CU	Self-reported measure of CU frequency, by days of use over the preceding 30 days using the TLFB method<br>Self-reported measure of CU quantity over the previous 30 days using the TLFB method, quantified in individually standardized cannabis joint sizes	Baseline, posttx (6 wk), & 3-mo post-baseline assessment	- ITT analyses with multiple imputation procedures using the multivariate imputation by chained equations software package in R<br>- Cohen’s *d* ES was used to calculate changes from baseline to follow-up (within-group ES), and between the 3 groups	6-wk follow-up: 62.8% (*n* = 361/575)<br>3-mo follow-up: 75.1% (*n* = 432/575)	- All groups exhibited reduced CU days after 3 mo (social presence: –8.2 days; service team: –9.8 days; internet as usual: –4.2 days)<br>- Participants in the service team group (*p* = 0.01; *d* = 0.60) reported significantly fewer CU days than those in the CG; the reduction of CU in the social presence group was not significant (*p* = 0.07; *d* = 0.40)<br>Upon review, the number of standard joints was deemed unreliable as a measurement and was dropped from analysis

	team (impersonal service team)<br><br>**CG:** Waiting list (access to internet as usual)						
Schaub et al., 2015	**IGs:**<br><br>***Group 1:*** Web-based self-help intervention with tailored chat counseling to reduce CU (CANreduce 1.0)<br>***Group 2:*** CANreduce 1.0 without tailored chat counseling<br>**CG:** Waiting list	CU	- Self-reported measure of CU frequency, by number of days used for the preceding 7 days, derived from the consumption diary<br><br>- Self-reported measure of CU quantity, by number of standardized cannabis joints consumed for the preceding 7 days, as specified in the consumption diary	Baseline & 3-mo post-baseline assessment	- Depending on the scale of the corresponding outcome, Mann-Whitney U tests, chi-square tests, or ANOVA were calculated<br>- Results from the imputed dataset (*n* = 50) were cross-checked with the non-imputed dataset in the latter analyses	62.0% (*n* = 191/308)	- Change in the mean number of CU days per wk at 3 mo differed between group 1 and group 2 (*B* = -0.75; *SE* = 0.32; t = -2.39; *p* = 0.02; *d* = 0.34; 95%CI [0.07, 0.61]), as well as between group 1 and CG (*B* = 0.70; SE = 0.32; t = 2.16; *p* = 0.03; *d* = 0.20; 95%CI [-0.07, 0.47])<br>- No differences between group 2 and CG (*B* = -0.05; SE = 0.33; t = -0.16; *p* = 0.87, *d* = -0.14; 95%CI [-0.43, 0.14])
**Study of *Grassessment: Evaluate Your Use of Cannabis***							
Copeland et al., 2017	**IG:** Web-based, self-complete motivational enhancement intervention for cannabis users that provides individualized extended feedback regarding use, motives, and harms (*Grassessment: Evaluate Your Use of Cannabis* + extended feedback)<br>**CG:** Screening + brief PNF (brief feedback version of the *Grassessment* program)	CU	- Self-reported measure of CU frequency, by number of days in the previous mo using the TLFB method<br><br>- Self-reported measure of CU quantity, by number of standard cannabis units (3 cones or 1 regular sized joint) in the previous mo using the TLFB method	Baseline & 1 mo post-baseline assessment	- Wilcoxon signed rank tests were performed to examine short term changes in CU outcomes from baseline to follow up for all participants<br>- GEE approach was used to examine the relationship between the length of feedback received and changes in outcome variables	32.4% (*n* = 93/287)	- Participants in the brief feedback group significantly reduced their frequency of CU by a median of 6 days in the mo prior to follow up and those in the extended feedback group significantly reduced their CU by a median of 10 days<br>- Past month CU quantity was also significantly reduced among participants in both groups, with those in the brief feedback group reducing their use by a median of 17 standard cannabis units and the extended feedback group reducing their use by a median of 28 standard cannabis units<br>- Results from the GEE analyses did not find short term changes in CU frequency and quantity to be associated with intervention condition (i.e., the length of feedback received)
**Studies of *The Marijuana eCHECKUP TO GO (e-TOKE)* (*n* = 5)**							
Elliott et al., 2012	**IG:** Brief, norm-correcting, web-based prevention	CU	Self-reported measure of marijuana use/initiation in the previous mo, reported in %	Baseline & 1-mo post-	- Two-group one-way ANOVAs (or	1.6% (*n* = 4/245)	- 5.5% (*n* = 6/109) of participants in the IG reported initiating marijuana use in the intervening mo, while 11.4% (*n* = 15/132) of

	and intervention program for individuals currently abstaining from marijuana (The Marijuana eCHECKUP TO GO [e-TOKE] for Universities and Colleges)<br>**CG:** Assessment only		using/initiating in past mo (i.e., participants were asked to report on marijuana use/initiation by stating whether they had used in the previous mo)	baseline assessment	ANCOVAs if baseline differences existed)<br><br>- Chi-square tests		participants in the CG used marijuana during that mo<br>- Although this difference in proportion corresponds to a small-to-medium effect size (*d* = 0.38), it was not statistically significant (OR = 0.53; *SE* = 0.25; *z* = -1.33; *p* = 0.18; 95%CI [0.21, 1.35])
Elliott et al., 2014	**IG:** Brief, self-paced, web-based marijuana educational program that incorporates personalized feedback and norm correction (The Marijuana eCHECKUP TO GO [e-TOKE] for Universities and Colleges)<br>**CG:** Assessment only	CU	Self-reported measure of marijuana-use days over the past mo	Baseline & 1-mo post-baseline assessment	- ANOVAs<br><br>- Nonlinear transformations were used due to non-normal data	1.6% (*n* = 5/317)	Non-significant difference between IG and CG from baseline to 1-mo follow-up (*p* = 0.7353):<br><br>- Within effects (*d*): IG = 0.09; CG = 0.02<br><br>- Between-groups effect (*d*) = 0.08
		Cannabis-related consequences	Self-reported measure of marijuana-related problems in the past mo using the 18-item Rutgers Marijuana Problems Index (RMPI) (White, Labouvie, & Papadaratsakis, 2005)		- Cohen's *d* values were calculated for within- and between-group effects		Non-significant difference between IG and CG from baseline to 1-mo follow-up (*p* = 0.8067):<br><br>- Within effects (*d*): IG = -0.16; CG = -0.27<br><br>- Between-groups effect (*d*) = 0.10
Goodness et al., 2020	**IG:** Electronic screening and brief intervention to reduce marijuana use and consequences with a booster session (additional feedback on CU at the 3-mo timepoint following assessment) (eCHECKUPTOGO-cannabis + booster intervention)<br>**CG:** Baseline assessment + minimal general health information	CU	Self-reported measure of CU frequency, by days of use over the past 90 days accompanied by a 90-day calendar	Baseline, 3-, & 6-mo postbaseline assessment	Conditional LGM was performed to provide ES estimates for the influence of the intervention on 6-mo cannabis outcomes, using Cohen's general guidelines for f^2	3-mo follow-up: 8.2% (*n* = 4/49)<br><br>6-mo follow-up: 8.2% (*n* = 4/49)	Small-to-medium effects of intervention condition on CU frequency at 3 mo (f^2 = 0.10; *B* = −9.64; *SE* = −0.16; *p* = 0.03), and over the 6-mo timeframe (f^2 = 0.09; *B* = −9.30; *SE* = 0.27; *p* = 0.10)
		Cannabis-related consequences	Self-reported measure of cannabis negative consequences over the past 90 days using the 19-item Marijuana Problems Scale (MPS) (Stephens, Roffman, & Simpson, 1994)				Intervention did not influence cannabis-related consequences over the course of 3-mo (f^2 = 0.022; *B* = −6.54; SE = −0.10; *p* = 0.27); and similar findings were observed over 6 mo (f^2 = 0.002; *B* = 0.11; SE = 0.03; *p* = 0.86)

Palfai et al., 2014	**IGs:**<br>***Group 1(on-site[b]):*** Web-based screening and brief intervention for marijuana users (The Marijuana eCHECKUP TO GO [e-TOKE]<br>***Group 2 (off-site[b]):*** Marijuana eCHECKUP TO GO [e-TOKE]<br>**CGs:**<br>***Group 1 (on-site[b]):*** Baseline assessment + feedback on general health-related behaviors[c]<br>***Group 2 (off-site[b]):*** Baseline assessment + feedback on general health-related behaviors	CU<br><br>Cannabis-related consequences	Self-reported measure of smoked marijuana-use days in the last 90 days, accompanied with a 3-mo calendar<br><br>Self-reported measure of marijuana-related consequences over the past 90 days using the 19-item MPS	Baseline, 3-, & 6-mo postbaseline assessment	- Conditional LGM was used to provide ES estimates for the influence of intervention on outcomes<br>- Robust maximum likelihood estimator was used to accommodate missing data in all models (and because data were not normally distributed)<br>- Stratified analyses by site (on-site vs. off-site) were conducted to provide information about whether ES varied by context	3-mo follow-up: 10.6% (*n* = 13/123)<br><br>6-mo follow-up: 5.7% (*n* = 7/123)	- The frequency of marijuana use changed little over time for either IG (on-site + off-site); LGM indicated both non-significant change for the group as a whole<br>- Stratified analyses: small effect of intervention on marijuana use for the off-site subsample ($f^2 = 0.015$; *B* = 1.25; SE = 0.66; *p* = 0.06)<br>- The ES estimate for the influence of the intervention (on-site + off-site) on marijuana-related consequences over time suggested a small intervention effect, but not statistically significant ($f^2 = 0.04$; *B* = 0.66; *SE* = 0.53); *p* > 0 .05)<br>- Stratified analyses: medium effect of intervention on marijuana-related consequences for the on-site subsample (f^2 = 0.12; *B* = 1.25; *SE* = 0.66; *p* = 0.06)
Riggs et al., 2018	**IG:** Web-based marijuana use intervention providing university-specific personalized feedback with normative information and protective behavioural strategies (Adapted version of eCHECKUP TO GO)<br>**CG:** Healthy stress management	CU<br><br>Cannabis-related consequences	Self-reported measure of marijuana use, using five indicators: 1) hours high per wk (range = 0–168); 2) hours high per using day (range = 0–24); 3) days high per wk (range = 0–7); 4) wk high per mo (range = 0–4); and 5) periods high per wk (range 0–28)<br><br>Self-reported measure of marijuana-use consequences, assessed by summing the total number of consequences experienced in the last mo and the average severity of the endorsed consequences, on a 5-	Baseline & 6-wk post-baseline assessment	- GLMs tested direct program effects on marijuana use, and use consequences<br>- Multi-group GLMs then tested the moderating effect of sex on direct intervention effects	23.8% (*n* = 71/298)	- Participants in the IG showed significantly greater decreases in marijuana use at 6-wk posttest compared with participants in the CG including hours high per wk (*B* = -3.26; *SE* = 1.29; t = -2.53; *p* < 0.05 ; $\eta^2 = 0.03$), days high per wk (*B* = -0.75; *SE* = 0.19; t = -4.02; *p* < 0.01; $\eta^2 = 0.07$), wk high per mo (*B* = -0.37; *SE* = 0.11; t = -3.53; *p* < 0.01; $\eta^2 = 0.05$), and periods high per wk (*B* = -1.11; *SE* = 0.54; t = -2.06; *p* < 0.05; $\eta^2 = 0.02$) at follow-up than the CG; but not significant for hours high per using day (*B* = -0.1; *SE* = 0.31; t = -0.34; $\eta^2 = 0.00$)<br>- Partial η^2 effect sizes for significant differences were in the small to medium range Non-significant difference between IG and CG at 6-wk posttest, including number of consequences (*B* = -0.01; *SE* = 0.06; t = -0.10; $\eta^2 = 0.00$), and consequence severity (*B* = -0.67; *SE* = 0.80; t = -0.82; $\eta^2 = 0.00$)

			point scale from “never” to “always”				
**Study of *Ma réussite, mon choix***							
Côté et al., 2018	**IG:** Web-based tailored intervention to reduce CU<br>**CG:** Assessment only	CU	Self-reported measure of CU frequency (number of events) over the past mo on a 4-point rating scale: (1) never; (2) several times, but not every wk; (3) several times a wk, but not every day; and (4) every day	Baseline & posttx (2 mo)	ITT analysis using a last-observation-carry- forward strategy was used to impute missing data at 2 mo	19.0% (*n* = 112/588)	At 2 mo posttx, a higher proportion of participants in the IG had reduced their CU compared with the CG (10.8% vs 5.1%, *p* = 0.007)
**Studies of *Quit the Shit (QTS)* (*n* = 2)**							
Jonas et al., 2018	**IG:** Therapist-guided internet intervention for cannabis users with chat-based (synchronous) and time-lagged (asynchronous) counseling<br><br>(regular version of QTS, 50 days with chat)<br><br>**Comparators:**<br><br>***Group 1:*** QTS version 2, chat, and 28 days<br>***Group 2:*** QTS version 3, no chat, and 50 days<br>***Group 3:*** QTS version 4, no chat, and 28 days	CU	- Self-reported measure of CU frequency, by days of use over the past 30 days using the TLFB method, and by number of CU events (i.e., the sum of joints, bongs, and other CU) derived from the input from the TLFB form<br><br>- Self-reported measure of cannabis quantity in grams; participants were asked to estimate this sum over the past 30 days	Baseline, & 3, 6, & 12 mo after randomizatio n	ITT analyses with a total of 50 imputations performed	3-mo follow-up: 52.8% (*n* = 282/534)<br><br>6-mo follow-up: 61.8% (*n* = 330/534)<br><br>12-mo follow-up: 74.7% (*n* = 399/534)	- Significant and strong time effects (i.e., within-group ES) indicate a great overall reduction of CU. The strongest reduction in the imputed dataset was found in the CU days ($B = −0.34$; CI [−0.45, −0.23]; $p < 0.001$; $d = 2.05$), followed by the number of use events ($B = −0.51$; CI [−0.68, −0.34]; $p < 0.001$; $d = 1.21$)<br>- None of the 3-way interactions (chat × intervention length × time) on the cannabis-related outcomes were significant, suggesting no relevant effectiveness differences between program versions

Tossman et al., 2011	**IG:** Web-based counselling program developed to help young people to quit or reduce their CU significantly (original version of QTS)<br>**CG:** Waiting list	CU	- Self-reported measure of CU frequency, by number of days used in the last 30 days using the TLFB method<br><br>- Self-reported measure of CU quantity, by grams consumed in the last 30 days using the TLFB method	Baseline & 3-mo post-baseline assessment	Per-protocol (i.e., only includes the data of those who left valid follow-up data) and ITT analyses	52.5% (*n* = 678/1292), including 38.3% of lost to follow-up (*n* = 495/1292) and 14.2% of intervention dropouts (*n* = 183/1292)	- In the per-protocol analysis, participants in the IG showed a significantly stronger reduction in CU than participants in the CG, for both consumption measures<br>- Moderate-to-large between-group ES in use frequency ($d = 0.98$; $p < 0.001$) and quantity ($d = 0.75$; $p < 0.001$) were observed
**Study of *Reduce Your Use: How to Break the Cannabis Habit***							
Rooke et al., 2013	**IG:** Fully self-guided web-based treatment program for CU and related problems<br>**CG:** 6 modules of web-based educational information on cannabis	CU	- Self-reported measure of CU, by number of smoking days in the past mo using the TLFB method<br><br>- Self-reported measure of past-mo quantity of CU, by measuring standard cannabis units (SCUs), where a regular-sized joint or 3 cones equals 1 SCU<br>- Past-mo abstinence	Baseline, posttx (6 wk), & 3-mo post-baseline assessment	- CACE analyses with an EM imputation procedure for missing data (primary analysis)<br><br>- ITT analysis employing between-groups repeated measures ANOVA and using EM imputation	6-wk follow-up: 33.8% (*n* = 76/225)<br><br>3-mo follow-up: 45.8% (*n* = 103/225)	- At 6 wk, the IG reported significantly fewer days of CU during the past mo ($B = 3.82$; SE = 1.67; $p = 0.02$; $d = 0.38$)<br>- Findings at 3 mo were similar ($B = 5.37$; SE = 2.36; $p = 0.02$; $d = 0.31$)<br>- Significantly lower past-mo quantity of CU in the IG relative to the CG at the 6-wk post assessment ($B = 24.86$; SE = 9.78; $p = 0.01$; $d = 0.34$)<br>- At the 3-mo follow-up, past-mo quantity of cannabis consumed no longer differed significantly between groups ($B = 11.84$; SE = 8.45; $p = 0.16$; $d = 0.19$)<br>- At the 6-wk posttx assessment, the IG had a higher rate of abstinence (9.3%; *n* = 7/76) than did the control group (4.7%; *n* = 3/73), though the numbers were small and the difference not statistically significant (OR = 2.53; $p = 0.10$)<br>- Likewise, at the 3-mo follow-up, past-mo abstinence was higher in the IG (12.4%; *n* = 8/64) compared with the CG (6.6%; 4 out of 58), with the difference missing the conventional threshold of statistical significance (OR = 2.50; $p = 0.06$)
**Studies of social media-delivered cannabis interventions (*n* = 2)**							
Bonar et al., 2022	**IG:** Social media-based, MI and cognitive-behavioral intervention targeting CU among emerging adults<br>**CG:** Attention control	CU	- Self-reported measure of CU frequency, by total number of times used per day and total CU-days in the past 30-days across four modalities (i.e., smoking, vaping, dabbing, eating), using the TLFB method	Baseline, 3- (1 mo after the 8-wk intervention ended), & 6-mo	- Independent samples *t*-tests and chi-square analyses to compare groups on baseline demographics and cannabis consumption	3-mo follow-up: 9.3% (*n* = 14/149)<br><br>6-mo follow-up: 10.7% (*n* = 16/149)	- At 3-mo, the intervention and control groups both had 28% reductions in total times used cannabis, and the number of CU days declined 13.5% in the IG (CG = 10.8%); the IG reduced total estimated quantity by 7.9% (CG by 11.5%)<br>- At 6-mo the IG had reduced total frequency of CU by 30.1% (vs. CG increased by 6.8%),

			- Self-reported measure of CU quantity, by total quantity in the past 30-days across four modalities (i.e., smoking, vaping, dabbing, eating), using the TLFB method	postbaseline assessment	- Linear mixed effects models adjusted for baseline measurement of the outcome and balancing factors (i.e., age group, sex, recreational cannabis legality) to examine preliminary effects of the intervention on CU<br>- Two-sided test of the coefficient for the treatment/control indicator to test treatment effects, using estimated Cohen's *d* ES and 95% CI		and total days of CU by 19.2% (vs. 5.1% reduction in the CG); total estimated quantity in the IG reduced by 27.8% and the CG by 12.2%<br>- The only adjusted model with a statistically significant difference between groups involved total days of vaping, where the CG increased by 16.7% and the IG decreased by 43.5% (d = 0.40; 95% CI [0.05, 0.75]; *p* = 0.020)
Bonar et al., 2023	**IG:** Social media-delivered intervention for emerging adults who use cannabis that focused on physical activity, and include MI and CBT cannabis content intervention (physical activity + cannabis)	CU	Self-reported measure of CU frequency, by total number of times used and total CU-days in the past 30-days across four modalities (i.e., smoking, vaping, dabbing, eating), using the TLFB method	Baseline, 3- (1 mo after the 8-wk intervention ended), & 6-mo postbaseline assessment	- *t*-tests, ANOVA, and Chi-square analyses to compare groups on demographics, baseline cannabis consumption, and follow-up acceptability<br>- No formal statistical inference given the pilot nature of the study, thus (descriptive statistics based on follow-up completion over 3 mo and 6 mo)	3-mo follow-up: 11.7% (*n* = 7/60)<br><br>6-mo follow-up: 13% (*n* = 8/60)	- Overall CU days: IG (physical activity + cannabis) reduced days of use by 11.5% at 3 mo and increased 4.3% from baseline at 6 mo; CG 1 (physical activity only) decreased by 24.6% days at 3 mo and 32.0% days at 6 mo; and CG 2 (attention control) declined by 20.1% at 3 mo and 19.1% at 6 mo<br>- Number of times used: IG (physical activity + cannabis) reduced by 25.3% at 3 mo and 49.2% at 6 mo; CG 1 (physical activity only) decreased by 47.0% at 3 mo and 41.9% at 6 mo; and CG 2 (attention control) reduced by 36.0% at 3 mo and 36.3% at 6 mo
	**CGs:**<br>***Group 1:*** Physical activity only<br>***Group 2:*** Attention control	Cannabis-related consequences	Self-reported measure of past 3-mo cannabis-related consequences using the 26-item Brief Marijuana Consequences Questionnaire (B-MACQ) (Simons et al., 2012)				At 3 mo, changes in cannabis-related consequences were −10.5% (−19.2% at 6 mo) in the IG (physical activity + cannabis), −24.4% (−17.5% at 6 mo) in the CG 1 (physical activity only), and +1.5% (−7.8% at 6 mo) in the CG 2 (attention control)
**Studies of PFIs[c] (*n* = 4)**							
Buckner et al., 2020	**IG:** Online PFI for negative affect and cannabis (PFI-NAC)<br>**CG:** Assessment only	CU	Self-reported measure of CU frequency, by days of use over the preceding 2 wk using the TLFB method (participants reported the number of joints used per day, and CU frequency	Baseline & 2- wk post-baseline assessment	Analyses were conducted using a conditional process modeling program (PROCESS) that utilizes an ordinary least squares-based	38.1% (*n* = 24/63)	- The IG was related to less use than the CG at moderate (*B* = 1.74; *SE* = 0.73; *p* = 0.021; 95%CI [3.20, 0.27]) and higher levels of social anxiety (*B* = 3.37; SE = 1.10; *p* = 0.004; 95%CI [5.58, 1.16])<br>- At lower levels of social anxiety, intervention condition was unrelated to follow-up cannabis

			was calculated by auditioning the number of CU days)		path analytical framework to test for main and interaction effects		frequency, (*B* = 0.10; *SE* = 1.06; *p* = 0.924; 95%CI [2.23, 2.03)]
		Cannabis-related consequences	Self-reported number of cannabis problems using the 26-item B-MACQ (Simons et al., 2012)	Baseline only			At baseline, 77.8% endorsed at least one cannabis-related problem and 61.9% endorsed two or more problems
Cunningham et al., 2021	**IG:** Online, personalized normative feedback intervention + educational material about risky CU<br>**CG:** Educational materials about risky CU only	CU	Self-reported measure of CU frequency, by number of days used cannabis in the past 30 days	Baseline, & 3-, & 6-mo post-baseline assessment	Generalized linear mixed modelling framework, using an ITT approach	3-mo follow-up: 7.9% (*n* = 59/744)<br>6-mo follow-up: 9.1% (*n* = 68/744)	- Significant reductions in the number of days participants used cannabis over time across the sample as a whole (*p* < 0.001)<br>- No significant differences between intervention and control groups at 3- and 6-mo follow-ups for the number of days used cannabis in the last 30 days (*p* = 0.927)
		Cannabis-related consequences	Self-reported number of cannabis problems using the 19-item MPS	Baseline only	Descriptive statistics		NR
Lee et al., 2010	**IG:** Brief, web-based PFI for at-risk marijuana users transitioning to college<br>**CG:** Assessment only	CU	Self-reported measure of marijuana- or hashish-use days in the last 90 days using an adaptation of the Global Appraisal of Individual Needs–I (Dennis et al., 2002)	Baseline, & 3-, & 6-mo post-baseline assessment	- ANOVA to evaluate changes from baseline to 3-mo<br>- Cohen's *d* was selected as a common measure of ES	3-mo follow-up: 5.3% (*n* = 18/341)<br>6-mo follow-up: 6.2% (*n* = 21/341)	No significant time or time x intervention condition interactions in evaluating change in past 90-day use from baseline to 3-mo follow-up or change from baseline to 6-mo follow-up (data NR)
		Cannabis-related consequences	Self-reported measure of marijuana-related problems in the past mo using the 18-item RMPI				No significant time or time x intervention condition interactions in evaluating change in marijuana related problems from baseline to 3-mo follow-up or change from baseline to 6-mo follow-up (data NR)
Walukevich-Dienst et al., 2019	**IG:** Online PFI for cannabis-using college students with additional information on CU risks<br>**CG:** PNF only	CU	Self-reported measure of past-mo CU frequency using the Marijuana Use Form (MUF), measured on a 0–9 rating scale (0 = once per mo or less, 5 = 5–6 times per mo, 9 = 21 times per wk or more)	Baseline & 1-mo post-baseline assessment	- Differences between conditions and genders from baseline to follow-up were tested using ANOVA and chi-square analyses<br>- Moderation analyses were conducted using the PROCESS macro to test for main and interaction effects	11.9% (*n* = 27/227)	Main effects of intervention condition were non-significant (*B* = 0.389; *SE* = 0.548; t (172) = 0.710; *p* = 0.478; 95%CI [−0.692, 1.470]), as was the gender x intervention condition interaction (*p* = 0.839)
		Cannabis-related consequences	Self-reported measure of cannabis-related problems over the preceding 3 mo using a modified version of the 19-item				- Main effects of intervention condition were non-significant (*B* = 1.748; *SE* = 1.450; t (172) = 1.206; *p* = 0.230; 95%CI [−1.113, 4.610])

	MPS (an additional 13 items were included)		- Women (but not men) in the IG reported fewer cannabis-related problems at follow-up than women in the CG (*B* = −1.941; *SE* = 0.767; t (172) = −2.533; *p* = 0.012; 95% CI [−3.454, −0.428])

ANCOVA = repeated measures analysis of covariance; ANOVA = repeated measures analysis of variance; *B* = beta coefficient; CACE = complier average causal effect; CG = control group; CI = confidence interval; CU = cannabis use; *d* = Cohen's *d* effect-size coefficient (between-group difference); EM = expectation maximization; ES = effect size; f^2 = Cohen's f^2 effect-size coefficient within multiple regression model; GEE = generalized estimated equations; GLM = general linear model; IG = intervention group; ITT = intention-to-treat analysis; LGM = latent growth modeling; mo = month; MPS = Marijuana Problem Scale; NR = not reported; OR = odds ratio; *p* = p-value; PFI = personalized feedback intervention; PFI-NAC = personalized feedback intervention for negative affect and cannabis; posttx = posttreatment; NR = not reported; PNF = personalized normative feedback; SE = standard error; QTS = Quit the Shit; t = student t-test; TLFB = Timeline Follow-Back; vs. = versus; wk = week.

[a] Outcome variables of interest to this review (i.e., CU and cannabis-related consequences).

[b] Site variable refers to the location in which participants completed baseline assessment and intervention procedures; participants in the on-site condition completed procedures in a room at the health center while those in the off-site condition completed in a place of their choosing.

[c] No specific intervention name reported.
