# Supplementary material for: Digital Interventions for Recreational Cannabis Use Among Young Adults: Systematic Review, Meta-Analysis, and Behavior Change Technique Analysis of Randomized Controlled Studies
Source: J Med Internet Res. 2024 Apr 17;26:e55031. doi: 10.2196/55031 (PMC11063887; doi:10.2196/55031)

Multimedia Appendix 9. Risk-of-bias assessment of each included study for cannabis use and cannabis consequences.

Risk-of-bias assessment of included studies for cannabis consequence outcome (*n* = 10), summarized by risk-of-bias domain

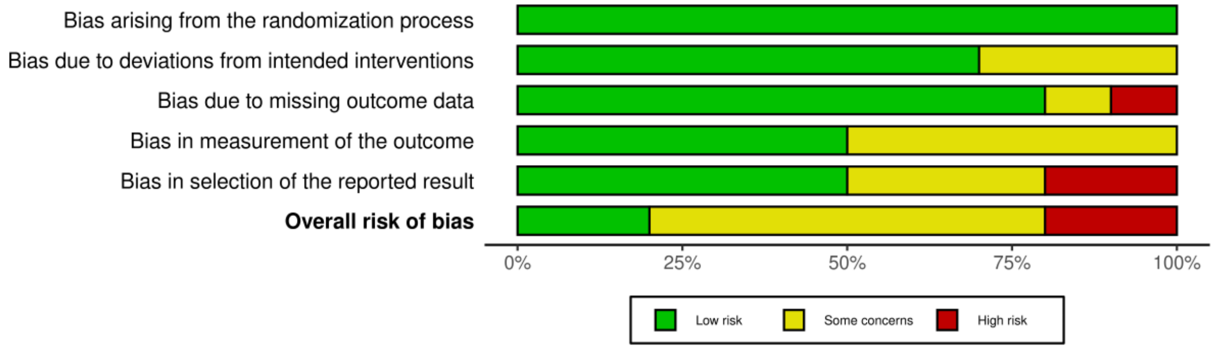

Summary of risk-of-bias assessments of each included study for cannabis consequence outcome (*n* = 10) at the outcome level, for each risk-of-bias domain

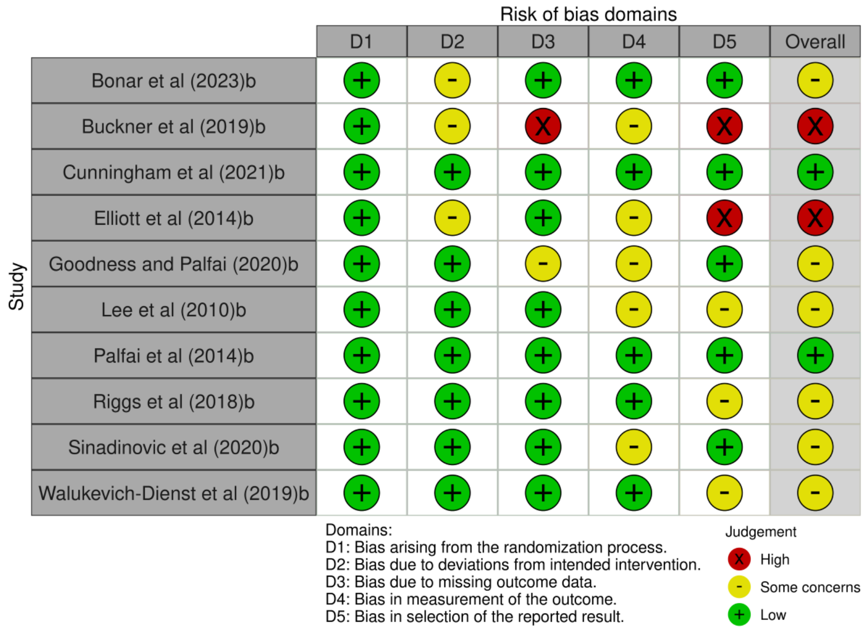

Risk-of-bias assessment of included studies for cannabis use outcome (N = 19), summarized by risk-of-bias domain

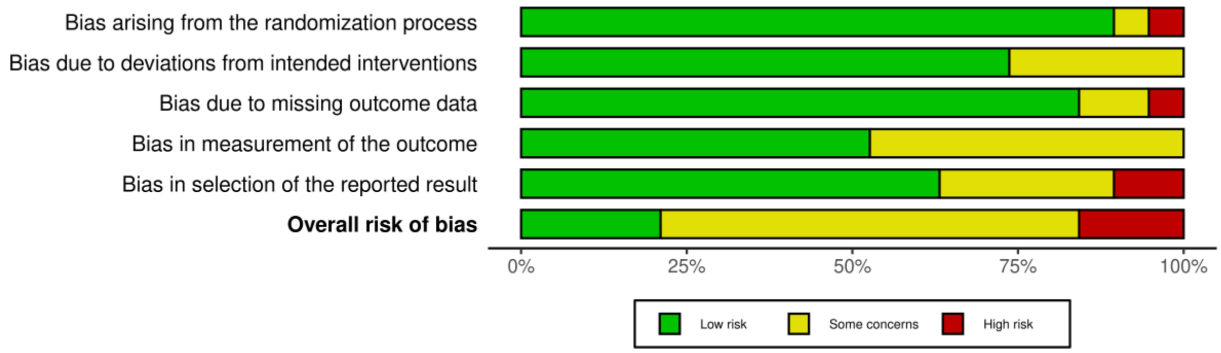

Summary of risk-of-bias assessments of each included study for cannabis use outcome (N= 19) at the outcome level, for each risk-of-bias domain

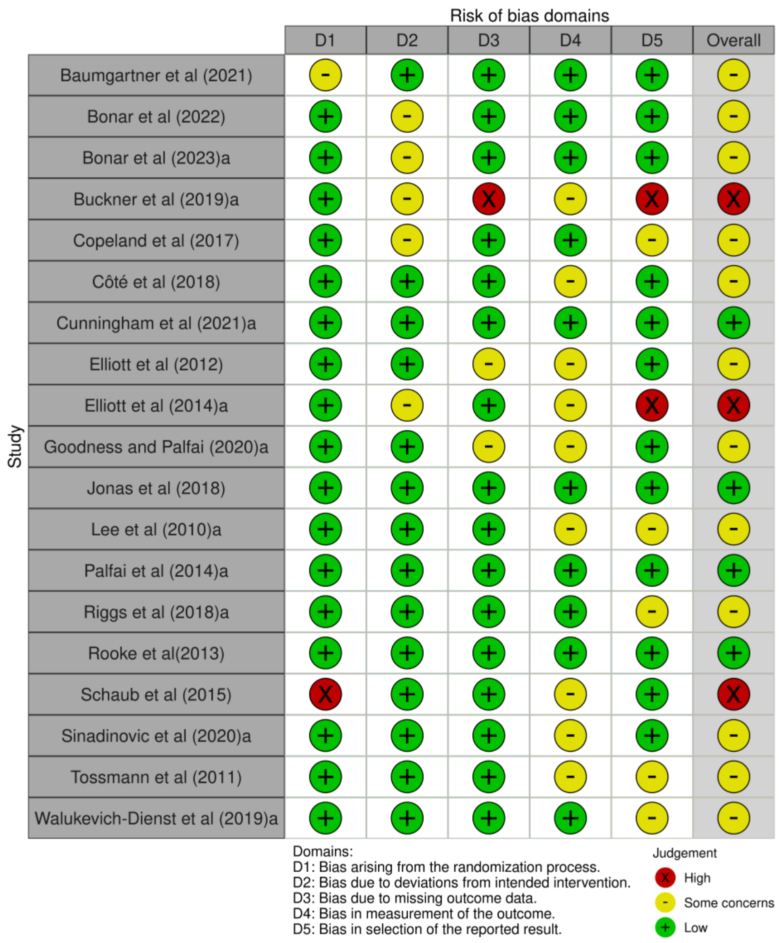

Supplement: Multimedia Appendix 9 [file jmir_v26i1e55031_app9.pdf]
