# Supplementary material for: Digital Interventions for Recreational Cannabis Use Among Young Adults: Systematic Review, Meta-Analysis, and Behavior Change Technique Analysis of Randomized Controlled Studies
Source: J Med Internet Res. 2024 Apr 17;26:e55031. doi: 10.2196/55031 (PMC11063887; doi:10.2196/55031)
Supplement: Multimedia Appendix 10 [file jmir_v26i1e55031_app10.pdf]

Multimedia Appendix D. Excluded studies and reasons for exclusion from the meta-analysis.

Cannabis use:

Bonar et al (2022): No mean difference reported.
Bonar et al (2023): No mean difference reported.
Buckner et al (2019): Mean (SD) rather than coefficient B, and data available for baseline and 2 weeks post, but nothing for 3 months.
Copeland et al (2017): No mean difference reported.
Côté et al (2018): Data presented in a dichotomous rather than continuous form (% n).
Cunningham et al (2021): No mean difference reported.
Elliott et al (2012): Data presented in a dichotomous rather than continuous form (% n).
Elliott et al (2014): Mean (SD) rather than coefficient B, and data available for baseline and 1 month post, but nothing for 3 months.
Goodness and Palfai (2020): Inadequate results - Negative SE sometimes and positive SE sometimes, poorly reported results.
Jonas et al (2018): Inadequate outcome measurement - Outcomes were measured at baseline, 3, 6, and 12 months after randomization using the Timeline Followback procedure. However, the Timeline Followback procedure only assessed consumption in the last 30 days. Therefore, we removed the study as it did not evaluate the same time frame as the others.
Lee et al (2010): Withdrawn due to the analyses conducted in the study - Included Family History and Contemplation in the analysis model.
Palfai et al (2014): No data at 3 months - We concluded that the coefficient B ([B= 1.25 (.66), p = .06]) was associated with the 6-month period due to this sentence: "As shown in Table 2, the frequency of marijuana use changed little over time for either intervention group. Indeed, unconditional latent growth models indicated both non-significant change for the group as a whole and provided little evidence for individual differences in change over the 6-month period."
Riggs et al (2018): Mean (SD) rather than coefficient B, and data available for baseline and 6 weeks post, but nothing for 3 months.
Schaub et al (2015): Inadequate results - the effect size is not within the confidence intervals, very small standard errors, which should indicate high significance, but this is not the case.
Sinadinovic et al (2020): Withdrawn due to the analyses conducted - Poisson model.
Walukevich-Dienst et al (2019): Mean (SD) rather than coefficient B, and data available for baseline and 1 month post, but nothing for 3 months.

Consequences:

Bonar et al (2023): No mean difference reported.
Buckner et al (2019): Mean (SD) rather than coefficient B, and data available for baseline and 2 weeks post, but nothing for 3 months.

Cunningham et al (2021): No mean difference reported.
Elliott et al (2014): Mean (SD) rather than coefficient B, and data available for baseline and 1 month post, but nothing for 3 months.
Goodness and Palfai (2020): Latent Growth Model with limited available data.
Lee et al (2010): Correlations, with missing data at 3 months and 6 months.
Palfai et al (2014): Mean (SD) rather than coefficient B, with missing N at baseline and 3 months.
Riggs et al (2018): Mean (SD) rather than coefficient B, and data available for baseline and 6 weeks post, but nothing for 3 months.
Sinadinovic et al (2020): Mean (SD) rather than coefficient B, and data available for baseline and 3 months could have been included if there were enough studies to pool.
Walukevich-Dienst et al (2019): Mean (SD) rather than coefficient B, and data available for baseline and 1 month post, but nothing for 3 months.
