# Supplementary material for: Digital Interventions for Recreational Cannabis Use Among Young Adults: Systematic Review, Meta-Analysis, and Behavior Change Technique Analysis of Randomized Controlled Studies
Source: J Med Internet Res. 2024 Apr 17;26:e55031. doi: 10.2196/55031 (PMC11063887; doi:10.2196/55031)
Supplement: Multimedia Appendix 11 [file jmir_v26i1e55031_app11.pdf]

Multimedia Appendix 11. Summary of evidence according to the Grading of Recommendations Assessment, Development, and Evaluation tool.

Certainty assessment							№ of patients		Effect	Certainty	Importance
№ of studies	Study design	Risk of bias	Inconsistency	Indirectness	Imprecision	Other considerations	Intervention	Control	Absolute (95% CI)		
**Cannabis use**											
3	Randomised trials	Serious[a]	Not serious	Not serious	Not serious	None	1368	729	SMD **6.79 SD lower** (9.59 lower to 4 higher)	⨁⨁⨁◯ Moderate	CRITICAL

CI: Confidence Interval; **SMD:** Standardised Mean Difference

Explanations

a. Baumgartner (2021): Significant differences between groups at baseline might suggest a problem with the randomization process. Tossmann (2011): Outcomes assessors were probably aware of the intervention assignment + No pre-specified analysis plan.
